# Efficacy and safety of Abelmoschus manihot capsule combined with ACEI/ARB on diabetic kidney disease: a systematic review and meta analysis

**DOI:** 10.3389/fphar.2023.1288159

**Published:** 2024-01-05

**Authors:** Ying Tan, Ziqi Zhang, Peipei Zhou, Qiling Zhang, Nan Li, Qianhua Yan, Liji Huang, Jiangyi Yu

**Affiliations:** ^1^ Department of Endocrinology, Jiangsu Province Hospital of Chinese Medicine, Affiliated Hospital of Nanjing University of Chinese Medicine, Nanjing, China; ^2^ The First Clinical Medical College, Nanjing University of Chinese Medicine, Nanjing, China

**Keywords:** diabetic kidney disease, Abelmoschus manihot, renin-angiotensin-aldosterone system inhibitors, systematic review, meta-analysis

## Abstract

**Background:** Diabetic kidney disease (DKD) is one of the most serious microvascular complications of diabetes, with the incidence rate increasing yearly, which is the leading cause of chronic kidney disease (CKD) and end-stage kidney disease. Abelmoschus Manihot capsule, as a proprietary Chinese patent medicine, is widely used for treating CKD in China. Currently, the combination of Abelmoschus Manihot (AM) capsule and renin-angiotensin-aldosterone system inhibitor (RASI) has gained popularity as a treatment option for DKD, with more and more randomized control trials (RCTs) in progress. However, the high-quality clinical evidence supporting its application in DKD is still insufficient.

**Aim of the study:** To comprehensively and systematically evaluate the efficacy and safety of AM capsule combined with RASI in the treatment of DKD.

**Materials and methods:** English and Chinese databases such as Pubmed, Cochrane Library, Embase, CNKI, SinoMed, WF, and VIP were searched to collect the RCTs of AM capsule in treatment of DKD. Then Two investigators independently reviewed and extracted data from the RCTs which met the inclusion criteria. The quality of the data was assessed using the Cochrane risk of bias assessment tool, and meta-analysis was performed using RevMan 5.4 software.

**Results:** 32 RCTs with a total of 2,881 DKD patients (1,442 in the treatment group and 1,439 in the control group) were included. The study results showed that AM capsule combined with RASI could be more effective in decreasing 24h-UTP [MD = −442.05, 95% CI (−609.72, −274.38), *p* < 0.00001], UAER [MD = −30.53, 95% CI (−39.10, −21.96), *p* < 0.00001], UACR [MD = −157.93, 95% CI (−288.60, −27.25), *p* < 0.00001], Scr [MD = −6.80, 95% CI (−9.85, −3.74), *p* < 0.0001], and BUN [MD = −0.59, 95% CI (−1.07, −0.12), *p* = 0.01], compared to using RASI alone. According to the subgroup analyses, the combination of AM and ARB seems to be more effective in reducing UAER than the combination of ACEI, and the addition of AM may achieve a more significant clinical effect on decreasing Scr for DKD patients with 24h-UTP>2 g or Scr>110–133 μmol/L and >133 μmol/L. Furthermore, no additional adverse reactions were observed in the combination group [OR = 1.06; 95%CI: (0.66, 1.69), *p* = 0.82].

**Conclusion:** Combining AM with RASI may be a superior strategy for DKD treatment compared to RASI monotherapy. However, due to significant heterogeneity, the results should be interpreted with great caution, and more high-quality RCTs with multi-centers, different stages of DKD, large sample sizes, and long follow-up periods are still needed to improve the evidence quality of AM for DKD in the future.

**Systematic Review Registration:** https://www.crd.york.ac.uk/PROSPERO/#recordDetails; Identifier CRD42022351422

## Graphical Abstract

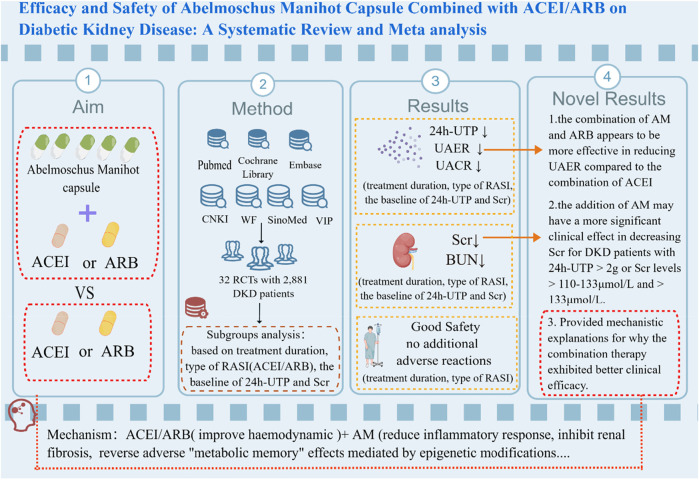

## 1 Introduction

According to the 10th edition of the International Diabetes Federation Atlas, the number of global adult diabetic patients has reached 537 million in 2021. It is expected to increase to 783 million by 2045 (Sun et al., 2022a), which indicates that more and more diabetic patients will develop diabetes complications over time. Diabetic kidney disease (DKD) is the most critical microvascular complication leading to disability and death of diabetes, accounting for approximately 40% of diabetes patients (Tuttle et al., 2014; Oshima et al., 2021), which is more likely to occur in East Asian people (Zoungas et al., 2014). Despite significant progress in DKD treatment strategies over the past three decades, patients with diabetes mellitus (DM) remain at a continuing high risk for CKD progression. DKD remains one of the leading causes of end-stage kidney disease (ESKD) and cardiovascular death, bringing stress to medical resources and the economy (Xie et al., 2018).

Renin-angiotensin-aldosterone system (RAAS) blocker medications combined with intensive metabolic control remain a mainstay of treatment for DKD patients, which is recommended by Chinese and foreign guidelines (Society, 2021; de Boer et al., 2022; ElSayed et al., 2023). Therefore, tolerated doses of RAAS inhibitors (commonly, angiotensin-converting enzyme inhibitors [ACEIs] and angiotensin-receptor blockers [ARBs]) have become the preferred first-line agents for clinicians treating DKD and are widely used in clinical practice. However, Non-Insulin-Dependent Diabetes With the Angiotensin II Antagonist Losartan (RENAAL) Study (Remuzzi et al., 2004), Irbesartan Diabetic Nephropathy Trial (IDNT) (Lewis et al., 2001) and STENO-2 Study (Gaede et al., 2016) all demonstrated that although proteinuria could be decreased after receiving ACEI/ARB treatment, it did not meet the needs of reducing proteinuria for most clinical DKD patients. Moreover, the risk of DKD progression remained high. Recently, sodium-glucose cotransporter 2 inhibitors (SGLT2i) and nonsteroidal mineralocorticoid receptor antagonists, as novel types of drugs for treating DKD, exhibited renal protective effects, and it should be noted that all studies evaluating the clinical efficacy of SGLT2i or nonsteroidal mineralocorticoid receptor antagonists were conducted on individuals undergoing ACEI/ARB therapy (ElSayed et al., 2023). However, no matter ACEI/ARB combined with SGLT2i or nonsteroidal mineralocorticoid receptor antagonist, both combinations can only delay renal function decline rather than reverse renal function, and all the average decrease in proteinuria is less than 400mg, which often fails to meet the clinical treatment needs of patients, especially those who have progressed to massive proteinuria or moderate to severe decline in renal function (Collaboration, 2020; Petrie et al., 2020; Tuttle et al., 2022; Bakris et al., 2023). Moreover, the clinical application of SGLT2i and nonsteroidal mineralocorticoid receptor antagonists both have restricted renal function and unavoidable adverse effects, thus limiting their use in DKD patients (Lin et al., 2021; Agarwal et al., 2022; Yang et al., 2022). Therefore, although Western medical treatments have been proven effective in slowing down the progression of kidney disease and alleviating renal endpoints, the worldwide prevalence DKD and its subsequent ESKD continues to increase. Consequently, there exists a significant demand to investigate renal protective medications that can reduce proteinuria and serum creatinine levels (Scr). As a result, an increasing number of DKD patients in China are turning to adding Traditional Chinese medicine (TCM) for treatment.

Traditional Chinese medicine (TCM) has a long tradition of treating CKD and is broadly used in clinical practice in Asian countries. As a representative drug of TCM for CKD therapy, Abelmoschus Manihot (https://www.worldfloraonline.org/taxon/wfo-0000510878) has been shown in clinical studies to decrease proteinuria, protect renal function, and have no significant side effects (Wei et al., 2023). Abelmoschus Manihot capsule (AM), also known as Huangkui capsule (HKC), is a proprietary Chinese medicine extracted from the Abelmoschus manihot flowers and has been approved by the State Food and Drug Administration of China (Z19990040) for the treatment of CKD since 1999 (Li et al., 2021). As early as 2011, DKD has replaced chronic glomerulonephritis as the primary cause of hospitalization and ESKD in China (Zhang et al., 2016). Multiple clinical studies have found that HKC combined with ACEI/ARB is more effective than ACEI/ARB alone in treating CKD. However, more attention has been paid to chronic glomerulonephritis (Dai et al., 2022) or IgA nephropathy (Jia et al., 2022) from these studies. In recent years, HKC combined with ACEI/ARB has been more widely used for treating DKD in clinical practice, and lots of novel data evaluating HKC in DKD have been published. However, the high level of evidence-based evidence remains indistinct because most clinical trials of HKC for the treatment of DKD only involved small samples. What’s more, inconsistent clinical results for reducing Scr and proteinuria between the combination of HKC and RASI and the use of RASI alone could be observed in different research. Therefore, this meta-analysis was conducted to investigate whether HKC combined with ACEI/ARB is more effective than using ACEI/ARB alone to treat DKD at different baselines of 24h-UTP and Scr and in different treatment durations. Furthermore, in most cases, ACEI and ARB are considered to have similar renal protective benefits (ElSayed et al., 2023) and risks, which are frequently used interchangeably in DKD treatments. However, to the best of our knowledge, no clinical study to date has focused on whether ACEI combined with HKC truly has the same clinical efficacy and safety as ARB combined with HKC for treating DKD. Therefore, this meta-analysis will also conduct a subgroup analysis on the type of RASI to provide more comprehensive and better guidance for clinical practice.

## 2 Materials and methods

This meta-analysis was conducted in accordance with the Preferred Reporting Items for Systematic Reviews and Meta-Analysis (PRISMA) (Moher et al., 2009) guidelines. The study was successfully registered on PROSPERO (CRD42022351422).

### 2.1 Database and search strategies

A comprehensive literature search of seven electronic databases was conducted: Pubmed, Cochrane Library, Excerpta Medica Database (Embase), China National Knowledge Infrastructure Database (CNKI), SinoMed, Wanfang Database (WF) and China Science and Technology Journal Database (VIP). The search period was from the establishment of the database until 4th, March 2023 with restricted language of Chinese and English. The search terms were “Abelmoschus”, “Huangkui”, “diabetic nephropathy”,“diabetic kidney disease” and so on. Different search strategy was applied for Chinese and foreign language databases. The details of the search strategy were available in the Supplementary Table S1.

### 2.2 Inclusion criteria

Studies were considered to be eligible for inclusion if they met criteria based on PICOS as follows: (1) Participants: the patients met the Kidney of Disease Outcomes Quality Initiative (KDOQI) clinical practice guidelines (Kdoqi, 2007) or Guidelines for the prevention and treatment of diabetic kidney disease (2021 version) in China (Society, 2021). (2) Intervention: the treatment group was treated Abelmoschus Manihot capsule combined with ACEI or ARB and basic treatment applied. (3) Comparison: the control group received the same ACEI or ARB and basic treatment as the treatment group. The basic treatment included comprehensive management of lifestyle, blood glucose, blood pressure, blood lipids, anti-infection measures, acid-base homeostasis, and other related aspects. (4) Outcome indicators: primary outcome measures comprising 24h-urine total protein (24h-UTP), urinary albumin excretion rates (UAER) and serum creatinine (Scr), regardless of treatment duration, baseline of 24h-UTP, baseline of Scr and type of RASI. Secondary outcome measures the adverse drug reaction rate based on treatment duration and type of RASI. (5) Study design: RCTs regardless of protocols, bias or blinding.

### 2.3 Exclusion criteria

Studies that met any of the following criteria would be excluded: (1) duplicated publications (only selecting the latest and most comprehensive data). (2) systematic review or meta-analysis, observational study, theoretical explorations, case reports, animal or cell experiments and studies involving patients with kidney damage relating to diseases other than DKD. (3) improper intervention including application of other TCM therapy between the two groups and other species of Abelmoschus, except for Abelmoschus Manihot. (4) no sufficient valid data be described. (5) no RCTs. (6) studies of low quality, such as flawed study design or inappropriate statistical methods.

### 2.4 Literature screening and data extraction

Two investigators independently screened the literature, extracted data, and cross-checked it. In case of divergence between the two investigators, comprehensive evaluation was conducted by a third senior investigator. If the data was missing or unavailable directly from the articles, we had tried to contact the corresponding author for obtaining relevant data. When selecting literature, we first read the title and abstract to exclude duplicates and significantly unrelated literature, and then read the full text further to determine whether it could be ultimately included.

The content of data extraction mainly included: (1) basic information of the included research, including research title, first author, year and journal of publication; (2) the baseline characteristics of the study subjects, including sample size, age and gender in each group; (3) specific details of intervention measures, such as dose and course of AM, type of ACEI/ARB; (4) key elements of bias risk assessment; (5) outcome indicators.

### 2.5 Evidence quality evaluation

Two researchers independently used the Cochrane Collaboration Handbook for Systematic Reviews of Intervention to evaluate the quality of the included RCTs from the following seven aspects: (1) random sequence generation; (2) allocation concealment; (3) blind methods for participants and researchers; (4) blinding of outcome assessment; (5) data integrity; (6) selective outcome reporting; (7) other bias sources. Bias risk of the studies was sorted into three levels: “high risk”, “low risk” and “unclear risk”. Divergences were resolved through discussion or a third senior investigator evaluation if necessary.

### 2.6 Statistical analysis

The statistical analyses were undertaken using Review Manager 5.4 software. The continuous variables were evaluated by mean differences (MD) with 95% confidence intervals (95% CIs), while the categorical variables were measured by odds ratios (ORs) and their 95% CIs. If the 95% CI for the OR includes 1.00, the OR is not statistically significant. I2 test was used to assess the heterogeneity of study results. If heterogeneity was low (I^2^ < 50%, *p* ≥ 0.1), the fixed-effect model was selected for the analysis; otherwise, the random-effect model was applied (I^2^ > 50%, *p* < 0.1). Meanwhile, we used subgroup analyses to evaluate potential moderating factors and explore the possible sources of heterogeneity. Funnel plots were used for assessing publication bias if more than ten studies were included in the analysis. Finally, a *p* value of <0.05 was considered statistically significant.

## 3 Results

### 3.1 Search results and study characteristics

**FIGURE 1:**
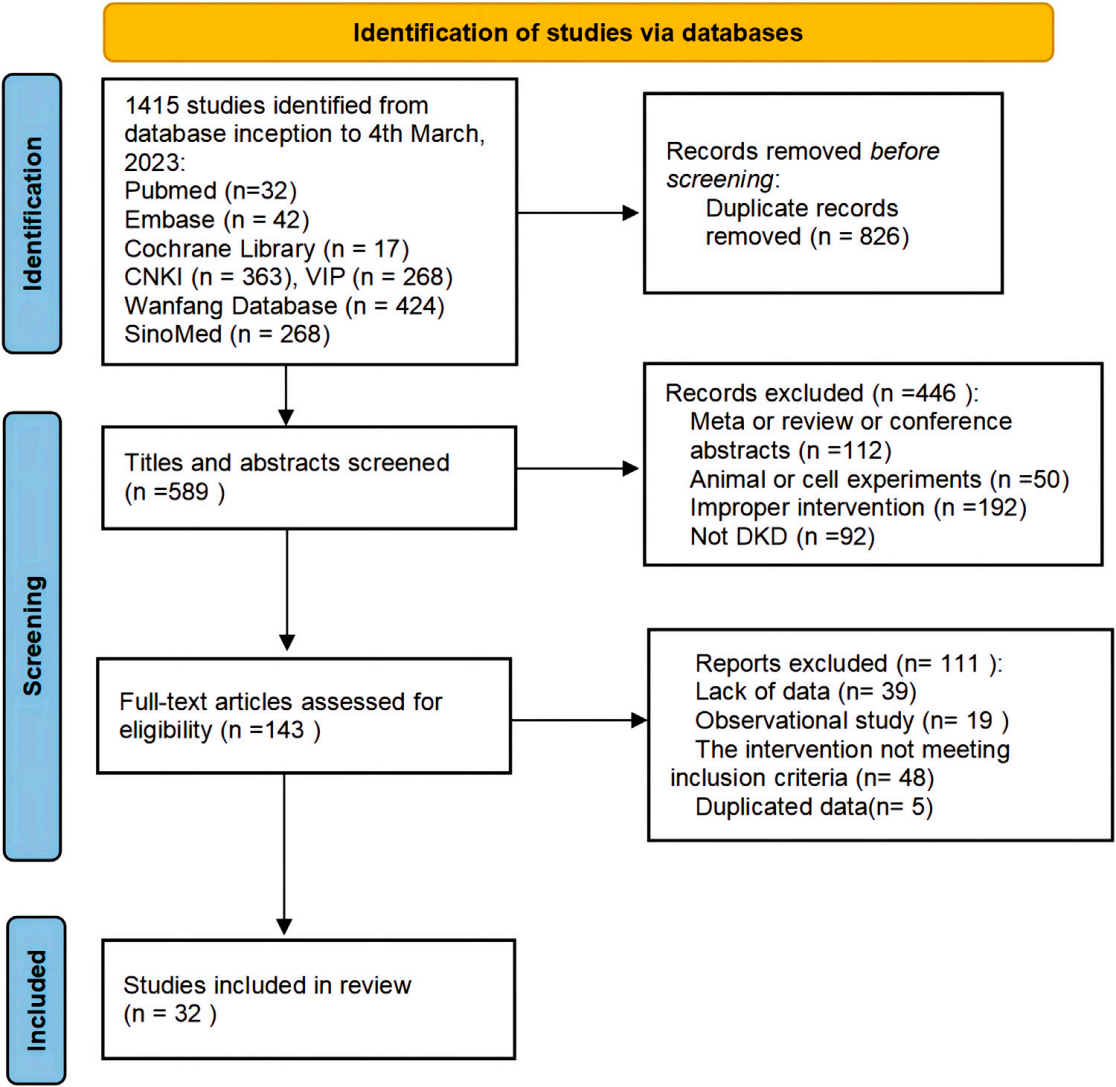
Flow diagram of studies selection process.

A total of 1,415 studies (1,323 in Chinese and 92 in English) were retrieved through database searching. After excluding 826 duplicates by EndNote and manually, 589 articles remained for further examination. After screening titles and abstracts, 446 studies were removed due to non-clinical trials, meta or reviews or conference abstracts, improper intervention and non-DKD. After reading the full text of 143 remaining articles, 111 articles were excluded for the following reasons: lack of data (*n* = 39), observational studies (*n* = 19), interventions not meeting the inclusion criteria (*n* = 48) and not duplicated data (*n* = 5). Finally, 32 studies were considered eligible for systematic evaluation. The study selection process is shown in Figure 1.

In total, 32 studies with 2,881 DKD patients (1,442 from the treatment group and 1,439 from the control group) were included for the meta-analysis. All the selected studies were published from 2010 to 2022, with a course of treatment ranging from 8 to 24 weeks. The specific characteristics of the included 32 studies are presented in Table 1.

**TABLE 1:** Basic characteristics of the included studies.

Study	Sample size	Gender (M/F)		Age(y)		Intervention		Duration	Outcomes
	(T/C)	T	C	T	C	T	C		
Cai et al. (2010)	25/25	12/13	13/12	45.66 ± 12.23	44.89 ± 12.75	AM(2.5 g,tid)+Control group treatment	Valsartan(80 mg,qd)+BT	8w	②④⑥
Chen et al. (2014)	75/75	38/37	39/36	58.1 ± 10.9	58.7 ± 10.1	AM(2.5 g,tid)+Control group treatment	Benazepril Hydrochloride Tablet(10 mg,qd)+BT	8w	③④⑤⑥
Dai et al. (2017)	40/40	19/21	18/22	46.21 ± 12.13	47.24 ± 11.18	AM(2.5 g,tid)+Control group treatment	Valsartan(80 mg,qd)+BT	12w	②④⑤⑥
Gao et al. (2017)	40/40	26/14	27/13	67.39 ± 4.72	67.83 ± 4.6	AM(2.5 g,tid)+Control group treatment	Valsartan(80 mg,qd)+BT	8w	②③④⑤
Gao (2017)	40/40	29/11	25/15	52.64 ± 9.15	54.68 ± 11.38	AM(2.5 g,tid)+Control group treatment	Irbesartan(150 mg,qd)+BT	16w	②③④⑤
Gu (2015)	100/100	57/43	53/47	69.4 ± 3.5	67.3 ± 5.9	AM(2.5 g,tid)+Control group treatment	Valsartan(80 mg,qd)+BT	8w	②③④⑤⑥
Huang et al. (2022)	40/40	NM	NM	NM	NM	AM(2.5 g,tid)+Control group treatment	Irbesartan(150–300 mg,qd)+BT	12w	①③④⑤
Jin and Qin (2018)	54/54	30/24	27/27	58.4 ± 5.9	55.4 ± 5.9	AM(2.5 g,tid)+Control group treatment	Valsartan(80 mg,qd)+BT	8w	④⑥
Ke (2020)	39/39	22/17	20/19	49.85 ± 9.03	50.42 ± 8.11	AM(2.5 g,tid)+Control group treatment	Valsartan(80 mg,qd)+BT	8w	②④⑤
Li (2019)	50/50	26/24	27/23	70.23 ± 3.32	70.14 ± 4.03	AM(2.5 g,tid)+Control group treatment	Valsartan(80 mg,qd)+BT	12w	②④⑤⑥
Li (2017)	31/31	20/11	19/12	60.56 ± 1.92	61.32 ± 1.74	AM(2.5 g,tid)+Control group treatment	Valsartan(80 mg,qd)+BT	8w	②
Li et al. (2016)	32/33	17/15	18/15	49.2 ± 18.2	49.3 ± 16.9	AM(2.5 g,tid)+Control group treatment	Benazepril Hydrochloride Tablet(10 mg,qd)+BT	16w	②③④
Li and Yun (2018)	30/30	16/14	18/12	58.21 ± 4.857	56.25 ± 6.013	AM(2.5 g,tid)+Control group treatment	Irbesartan(150 mg,qd)+BT	12w	③⑥
Lv and Yu (2015)	45/45	24/21	23/22	57.3 ± 3.1	56.9 ± 2.7	AM(2.5 g,tid)+Control group treatment	Valsartan(80 mg,qd)+BT	8w	②⑥
Qiao (2015a)	30/30	11/19	9/21	53.7 ± 6.15	51.87 ± 5.02	AM(2.5 g,tid)+Control group treatment	Benazepril Hydrochloride Tablet(10 mg,qd)+BT	12w	②④⑤
Qiao (2015b)	41/41	27/14	25/16	57.2 ± 3.6	56.6 ± 3.5	AM(2.5 g,tid)+Control group treatment	Enalapril(10 mg,qd)+BT	8w	②③④
Rao (2016)	29/29	18/11	17/12	41.3 ± 2.48	42.78 ± 3.01	AM(2.5 g,tid)+Control group treatment	Benazepril Hydrochloride Tablet(10 mg,qd)+BT	8w	③④⑤
Shen et al. (2011)	60/60	25/35	33/27	41.0 ± 14.2	42.0 ± 13.60	AM(2.5 g,tid)+Control group treatment	Telmisartan(80 mg,qd)+BT	8w	③④⑥
Sun et al. (2012)	45/45	29/16	28/17	62.34 ± 12.18	62.23 ± 11.99	AM(2.5 g,tid)+Control group treatment	Benazepril Hydrochloride Tablet(10 mg,qd)+BT	12w	③④⑤⑥
Tang (2017)	42/42	24/18	25/17	57.0 ± 9.5	56.1 ± 10.4	AM(2.5 g,tid)+Control group treatment	Valsartan(80 mg,qd)+BT	8w	②③④⑤⑥
Wang (2019a)	39/38	25/14	21/17	70.1 ± 2.1	66.5 ± 2.3	AM(2.5 g,tid)+Control group treatment	Valsartan(80 mg,qd)+BT	8w	②③④⑤
Wang (2019b)	34/31	18/16	16/15	64.9 ± 2.5	65.2 ± 2.4	AM(2.5 g,tid)+Control group treatment	Valsartan(80 mg,qd)+BT	12w	②④⑤⑥
Wang et al. (2017)	60/60	39/21	37/23	51.18 ± 12.67	50.66 ± 11.27	AM(2.5 g,tid)+Control group treatment	Valsartan(80 mg,qd)+BT	8w	②④⑥
Wu (2016)	24/24	16/8	15/9	56.7 ± 6.5	55.4 ± 6.2	AM(2.5 g,tid)+Control group treatment	Irbesartan(150–300 mg,qd)+BT	16w	③④⑥
Xu et al. (2018)	19/19	10/9	12/7	54.14 ± 10.26	54.72 ± 10.31	AM(2.5 g,tid)+Control group treatment	Benazepril Hydrochloride Tablet(10 mg,qd)+BT	56d (8w)	③④⑤
Xu et al. (2016)	62/62	36/26	34/28	43.8 ± 2.9	43.2 ± 3.4	AM(2.5 g,tid)+Control group treatment	Valsartan(80 mg,qd)+BT	24w	④⑤
Xu (2020)	28/28	17/11	16/12	53.2 ± 12.7	52.5 ± 12.6	AM(2.5 g,tid)+Control group treatment	Valsartan(80 mg,qd)+BT	12w	②④⑤⑥
Yao et al. (2015)	23/23	15/8	16/7	51.2 ± 5.6	52.2 ± 5.2	AM(2.5 g,tid)+Control group treatment	Irbesartan(75 mg,qd)+BT	8w	④⑤
Zeng (2013)	25/25	NM	NM	NM	NM	AM(2.5 g,tid)+Control group treatment	Losartan Potassium Tablets(50 mg,qd)+BT	12w	③④
Zhao (2015)	46/46	50/42		NM	NM	AM(2.5 g,tid)+Control group treatment	AM(2.5 g,tid)+Control group treatment	8w	②④⑥
Zhao et al. (2022)	138/138	NM	NM	NM	NM	AM(2.5 g,tid)+Control group treatment	Irbesartan+BT	24w	①
Zhao et al. (2011)	56/56	30/26	31/25	45.02 ± 13.18	48.64 ± 11.60	AM(2.5 g,tid)+Control group treatment	Benazepril Hydrochloride Tablet(10 mg,qd)+BT	8w	③④⑤⑥

T, treatment group; C, control group; AM, abelmoschus manihot; BT, basic treatment; NM, not mentioned; y, year; w, week; d, day; tid, three times a day; qd, once a day; NM, not mentioned; ①UACR; ②UAER; ③24h-UTP; ④Scr; ⑤BUN; ⑥: adverse reaction rate.

### 3.2 Quality assessment of included studies

**FIGURE 2:**
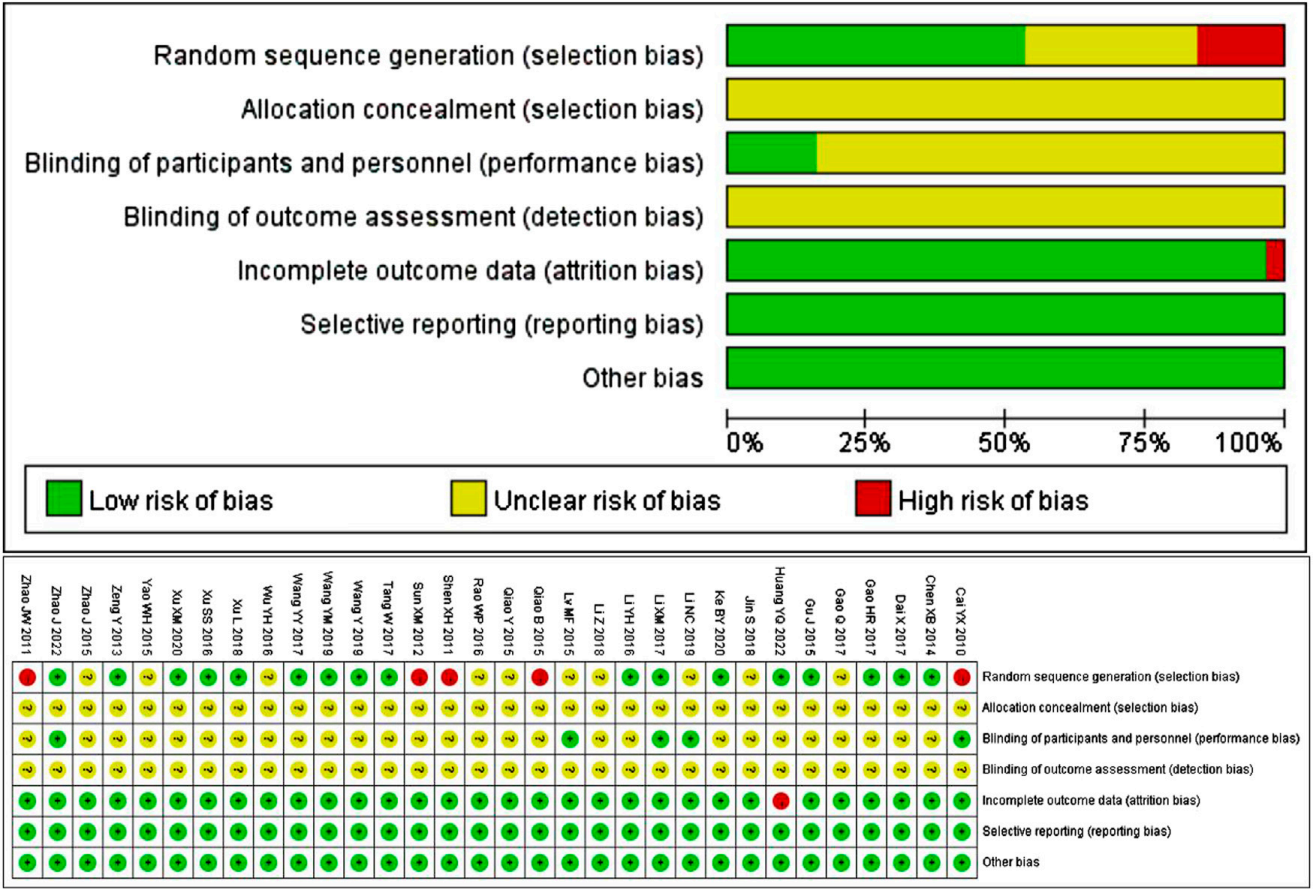
Risk of bias graph and risk of bias summary.

The quality of the 32 included studies was evaluated using the Cochrane Collaboration tool for assessing the risk of bias. 17 studies used reasonable random methods, including 16 studies using random number tables and 1 study using the red and blue ball lottery, and were evaluated as “low risk”. However, 5 studies used visit dates to make random sequence generation, which was inappropriate and recognized as “high risk”. The remaining 10 studies only stated randomization without specific details of stochastic methods, so they were considered “unclear risk”. None of the included studies mentioned whether the allocation sequence was concealed. Five studies explicitly used blind methods, with an assessment of low risk, while the rest of the studies didn’t mention blind methods, with an assessment of unclear risk. In terms of data, trials with complete results were judged to be low risk, except for one trial with one patient dropping out that was judged to be high risk. All 32 studies had clear outcome indicators, and there was no published bias or other biased information. The results of quality assessment are shown in Figure 2.

### 3.3 24h-UTP

**FIGURE 3:**
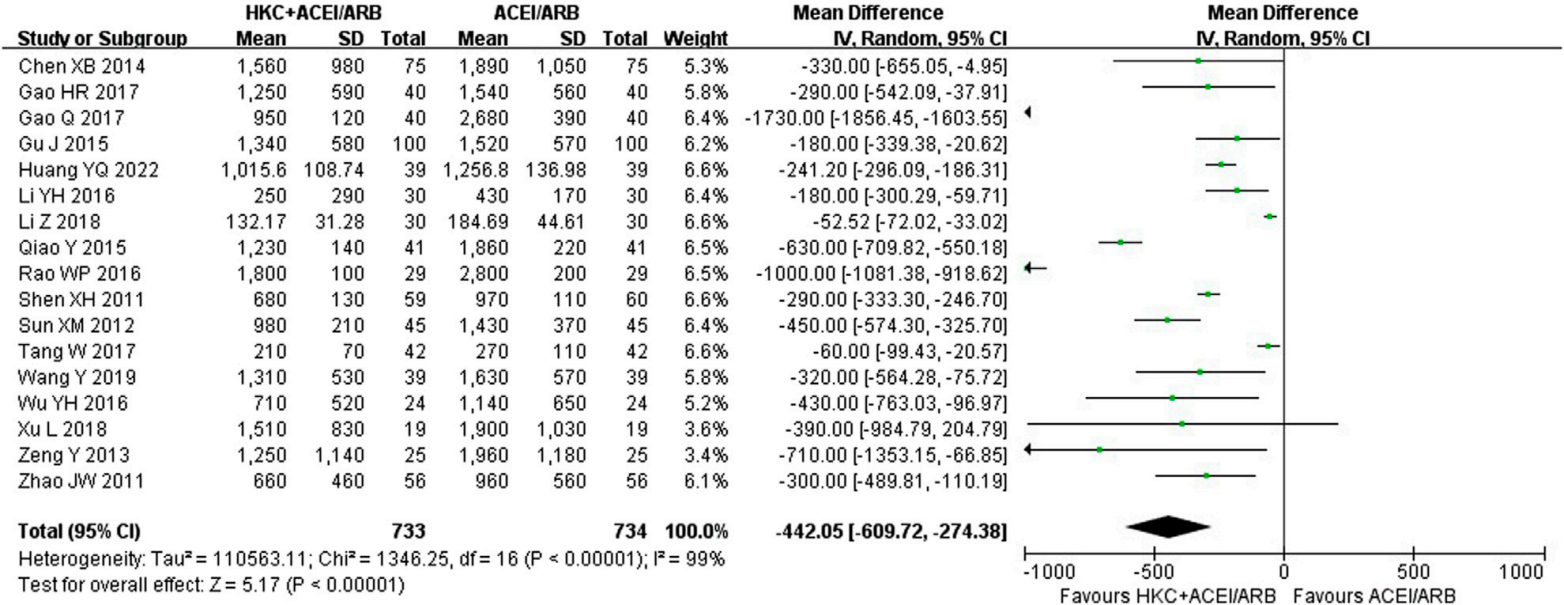
Forest plot of 24h-UTP.

Seventeen studies (Shen et al., 2011; Zhao et al., 2011; Sun et al., 2012; Zeng, 2013; Chen et al., 2014; Qiao 2015b; Gu, 2015; Li et al., 2016; Rao, 2016; Wu, 2016; Gao, 2017; Gao et al., 2017; Tang, 2017; Li and Yun, 2018; Xu et al., 2018; Wang 2019a; Huang et al., 2022) including 1,467 patients reported the 24h-UTP levels. As shown in Figure 3, those who added HKC in the experiment group exhibited statistically significant superiority in reducing 24h-UTP [MD = −442.05, 95% CI (−609.72, −274.38), *p* < 0.00001], but the heterogeneity was high (I^2^ = 99%, *p* < 0.00001). Thus, a random-effect model was used for statistical analysis.

Furthermore, we looked for the sources of high heterogeneity on 24h-UTP via subgroup analyses based on the treatment duration, type of RASI, baseline of 24h-UTP and baseline of Scr (Table 2; Supplementary Figures S1–S4). As shown in Table 2, the heterogeneity of patients in baseline of Scr 110–133 μmol/L and baseline of 24h-UTP 1–2 g obviously decreased. Interestingly, although treatment duration still had significant heterogeneity after subgroup analysis, the patients between the treatment group and control group presented no significant difference in the subgroup of treatment duration ≥16 weeks [MD = −782.68, 95%CI: (−1935.53, 370.17), *p* = 0.18].

**TABLE 2:** Subgroup analyses of 24h-UTP based on treatment duration, baseline of 24h-UTP, baseline of Scr and type of RASI.

Criteria for grouping	Subgroups	n	MD (95%CI)	I^2^(%)	Z	P
Treatment duration	8 weeks	10	−382.24(−601.85, −162.64)	98	3.41	0.0006
	12 weeks	4	−270.86(−457.87, −83.85)	96	2.84	0.005
	≥16 weeks	3	−782.68(−1935.53, 370.17)	99	1.33	0.18
Baseline of 24h-UTP	<1 g	3	−65.29(−101.21, −29.37)	53	3.56	0.0004
	1–2 g	7	−271.02(−303.06, −238.97)	0	16.58	<0.00001
	>2 g	7	−773.06(−1140.44, −405.67)	98	4.12	<0.0001
Baseline of Scr	<90 μmol/L	6	−568.39(−1019.54, −117.25)	99	2.47	0.01
	90–110 μmol/L	4	−200.80 (−352.74, −48.85)	95	2.59	0.01
	110–133 μmol/L	2	−343.80(−629.04, −58.56)	0	2.36	0.02
	>133 μmol/L	4	−651.13(−1042.86, −259.40)	95	3.26	0.001
Type of RASI	ACEI	7	−481.13(−740.83, −221.43)	96	3.63	0.0003
	ARB	10	−412.05(−599.69, −224.41)	99	4.30	<0.0001

RASI, Renin-angiotensin-aldosterone system inhibitor; ACEI, Angiotensin-converting enzyme inhibitor; ARB, Angiotensin-receptor blocker; 24h-UTP, 24h-urine total protein; Scr, Serum creatinine.

### 3.4 UAER

**FIGURE 4:**
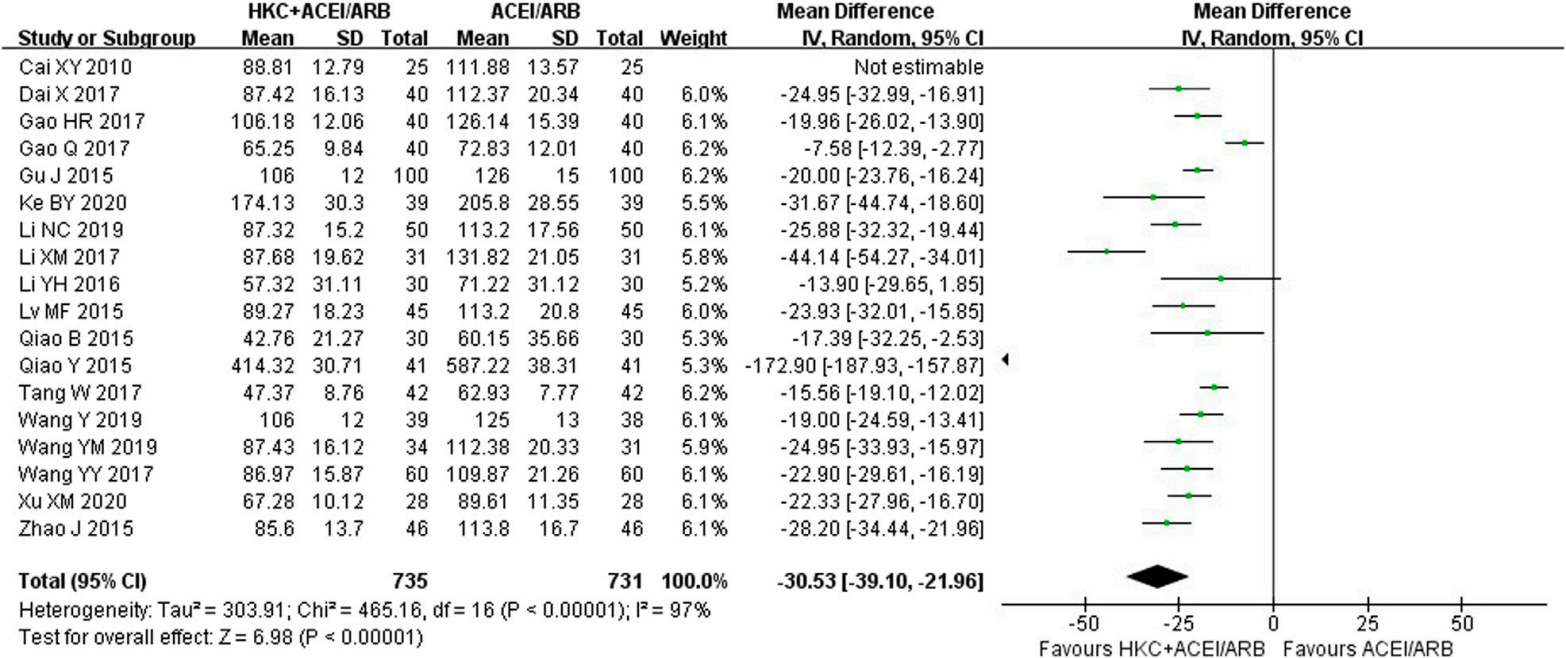
Forest plot of UAER.

Eighteen studies (Cai et al., 2010; Qiao 2015a; Qiao 2015b; Gu, 2015; Lv and Yu, 2015; Zhao, 2015; Li et al., 2016; Dai et al., 2017; Gao, 2017; Gao et al., 2017; Li, 2017; Tang, 2017; Wang et al., 2017; Wang 2019a; Wang 2019b; Li, 2019; Ke, 2020; Xu, 2020) encompassing 1,466 patients reported a significant reduction in UAER following the combination of HKC and RASI compared with RASI alone [MD = −30.53, 95% CI (−39.10, −21.96), *p* < 0.00001] (Figure 4). Because of high heterogeneity (I^2^ = 97%, *p* < 0.00001), a random-effect model was used to analyze the data.

Meanwhile, subgroup analyses of the treatment duration, baseline of 24h-UTP, baseline of Scr and type of RASI were performed to explore the sources of high heterogeneity (Table 3; Supplementary Figures S5–S8). The results showed that heterogeneity was reduced in the subgroups of treatment duration 12 weeks and ≥16 weeks, baseline of 24h-UTP <1g and 1–2 g, baseline of Scr 90–110 μmol/L and 110–133 μmol/L. With regard to the types of RASI, no statistical significance of reducing UAER was noted in the treatment of ACEI combined with HKC compared with ACEI alone [MD = −50.63, 95% CI (−125.86,24.61), *p* = 0.19]. Furthermore, the combination treatment also didn’t lead to a significant decrease of the UAER in the subgroup of baseline of 24h-UTP >2 g, compared with the control group [MD = −63.73, 95% CI (−173.96, 46.51), *p* = 0.26].

**TABLE 3:** Subgroup analyses of UAER based on treatment duration, baseline of 24h-UTP, baseline of Scr and type of RASI.

Criteria for grouping	Subgroups	n	MD (95%CI)	I^2^(%)	Z	P
Treatment duration	8 weeks	11	−33.06 (−44.41, −21.70)	97	5.70	<0.00001
	12 weeks	5	−23.88(−27.25, −20.51)	0	13.89	<0.00001
	≥16 weeks	2	−8.12(−12.72, −3.52)	0	3.46	0.0005
Baseline of 24h-UTP	<1 g	2	−15.48(−18.94, −12.03)	0	3.56	<0.00001
	1–2 g	3	−19.75(−22.52, −16.97)	0	16.58	<0.00001
	>2 g	2	−63.73(−173.96, 46.51)	100	7.35	0.26
Baseline of Scr	<90 μmol/L	7	−32.04(−49.77, −14.32)	98	3.54	0.0004
	90–110 μmol/L	7	−22.24 (−26.74, −17.74)	65	9.68	<0.00001
	110–133 μmol/L	2	−27.01(−32.79, −21.24)	0	9.17	<0.00001
Type of RASI	ACEI	3	−50.63(−125.86,24.61)	99	1.32	0.19
	ARB	15	−22.73(−26.35, −19.11)	81	12.31	<0.00001

RASI, Renin-angiotensin-aldosterone system inhibitor; ACEI, Angiotensin-converting enzyme inhibitor; ARB, Angiotensin-receptor blocker; 24h-UTP, 24h-urine total protein; Scr, Serum creatinine.

### 3.5 UACR

**FIGURE 5:**
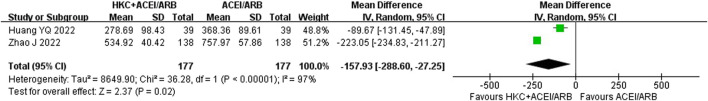
Forest plot of UACR.

After an analysis of two studies (Huang et al., 2022; Zhao et al., 2022) with a total of 354 patients that reported UACR, significant heterogeneity was found between studies (I^2^ = 97%, *p* < 0.00001). Due to high heterogeneity, a random-effect model was used and the analysis result showed that compared with the ACEI/ARB group, HKC combined with ACEI/ARB could reduce the UACR better [MD = −157.93, 95% CI (−288.60, −27.25), *p* < 0.00001] (Figure 5).

### 3.6 Scr

**FIGURE 6:**
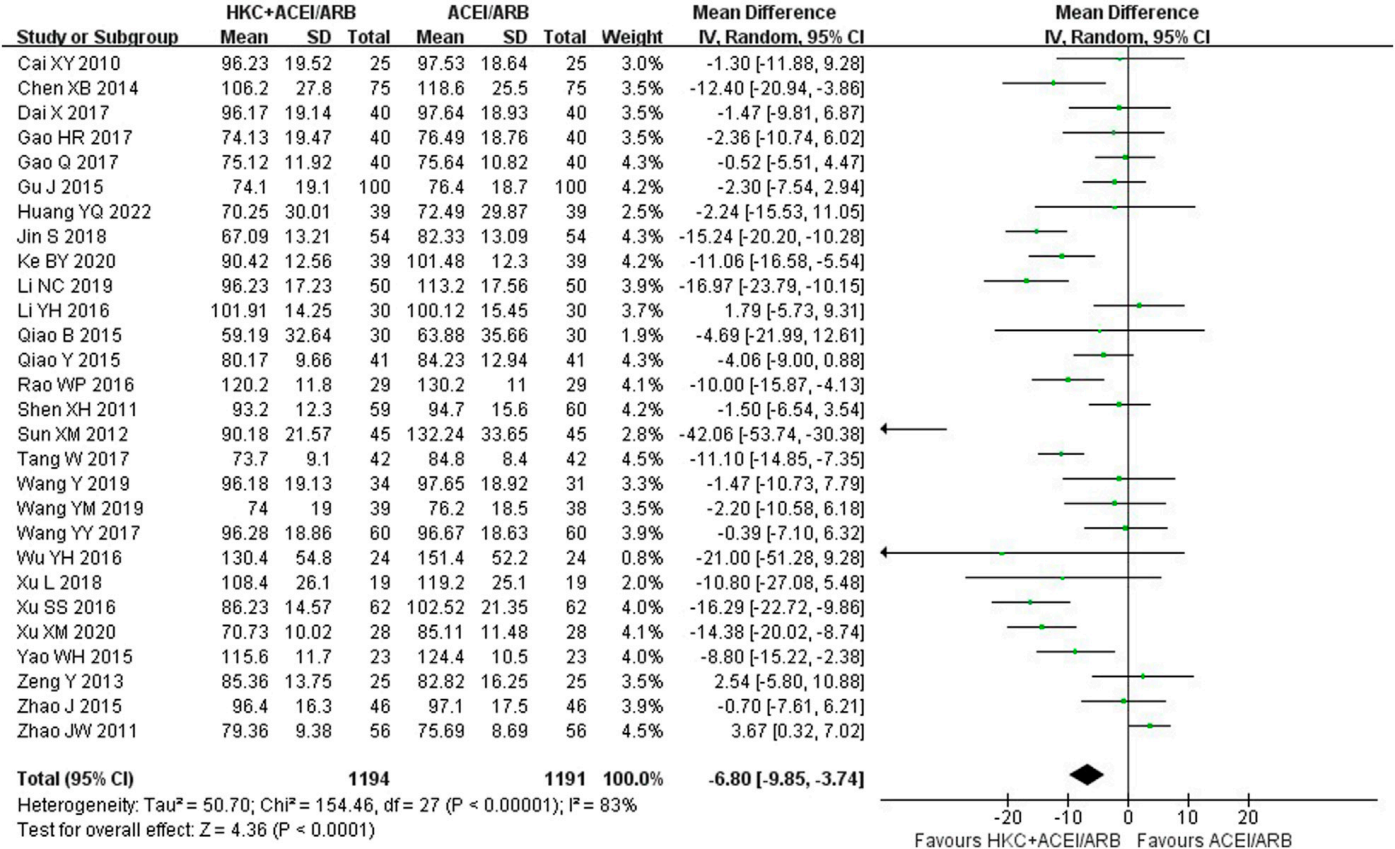
Forest plot of Scr.

Twenty-eight studies (Cai et al., 2010; Shen et al., 2011; Zhao et al., 2011; Sun et al., 2012; Zeng, 2013; Chen et al., 2014; Gu, 2015; Qiao 2015a; Qiao 2015b; Yao et al., 2015; Zhao, 2015; Li et al., 2016; Dai et al., 2017; Gao et al., 2017; Gao, 2017; Jin and Qin, 2018; Li, 2019; Ke, 2020; Huang et al., 2022; !!! INVALID CITATION !!!), comprising of 2,385 patients, contributed to this analysis and the results indicated that combination treatment significantly decreased the Scr level [MD = −6.80, 95% CI (−9.85, −3.74), *p* < 0.0001], with a random-effect model (I^2^ = 83%, *p* < 0.00001), (Figure 6).

Furthermore, subgroup analyses were conducted according to the following criteria for grouping: treatment duration, baseline of 24h-UTP, baseline of Scr and type of RASI (Table 4; Supplementary Figures S9–S12). The results showed that heterogeneity was obviously reduced in the subgroup of baseline of 24h-UTP 1–2 g (I^2^ = 26%, *p* = 0.23) and baseline of Scr 110–133 μmol/L (I^2^ = 0%, *p* = 0.54). Of note, although the heterogeneity of the two subgroup analyses significantly reduced, the patients in the subgroup analysis of baseline of 24h-UTP 1–2 g [MD = −0.32, 95% CI (−3.24,2.59), *p* = 0.83] presented no evidence of benefit for reducing the Scr level compared with control group. In addition, there was also no significant difference between the HKC + ACEI/ARB group and the ACEI/ARB group in the subgroup of treatment duration ≥16weeks [MD = −6.40, 95% CI (−16.66, 3.86), *p* = 0.22], baseline of 24h-UTP <1 g [MD = −5.08, 95% CI (−17.69, 7.52), *p* = 0.43], baseline of Scr 90–110 μmol/L [MD = −1.52, 95% CI (−5.72, 2.68), *p =* 0.48].

**TABLE 4:** Subgroup analyses of Scr based on treatment duration, baseline of 24h-UTP, baseline of Scr and type of RASI.

Criteria for grouping	Subgroups	n	MD (95%CI)	I^2^(%)	Z	*P*
Treatment duration	8 weeks	16	−5.55(−8.82 −2.28)	79	3.33	0.0009
	12 weeks	8	−10.13 (−18.86, −1.39)	87	2.27	0.02
	≥16 weeks	4	−6.40(−16.66, 3.86)	84	1.22	0.22
Baseline of 24h-UTP	<1 g	2	−5.08(−17.69, 7.52)	89	0.79	0.43
	1–2 g	7	−0.32 (−3.24,2.59)	26	0.22	0.83
	>2 g	7	−10.16 (−18.17,2.14)	88	2.48	0.01
Baseline of Scr	<90 μmol/L	8	−4.32(−8.06, −0.58)	55	2.26	0.02
	90–110 μmol/L	9	−1.52 (−5.72, 2.68)	77	0.71	0.48
	110–133 μmol/L	5	−11.99(−15.22, −8.77)	0	7.29	<0.00001
	>133 μmol/L	6	−15.82(−25.05, −6.59)	87	3.36	0.0008
Type of RASI	ACEI	8	−9.13(−17.18, −1.09)	90	2.22	0.03
	ARB	20	−6.26(−9.25, −3.27)	74	4.11	<0.00001

RASI, Renin-angiotensin-aldosterone system inhibitor; ACEI, Angiotensin-converting enzyme inhibitor; ARB, Angiotensin-receptor blocker; 24h-UTP, 24h-urine total protein; Scr, Serum creatinine.

### 3.7 BUN

**FIGURE 7:**
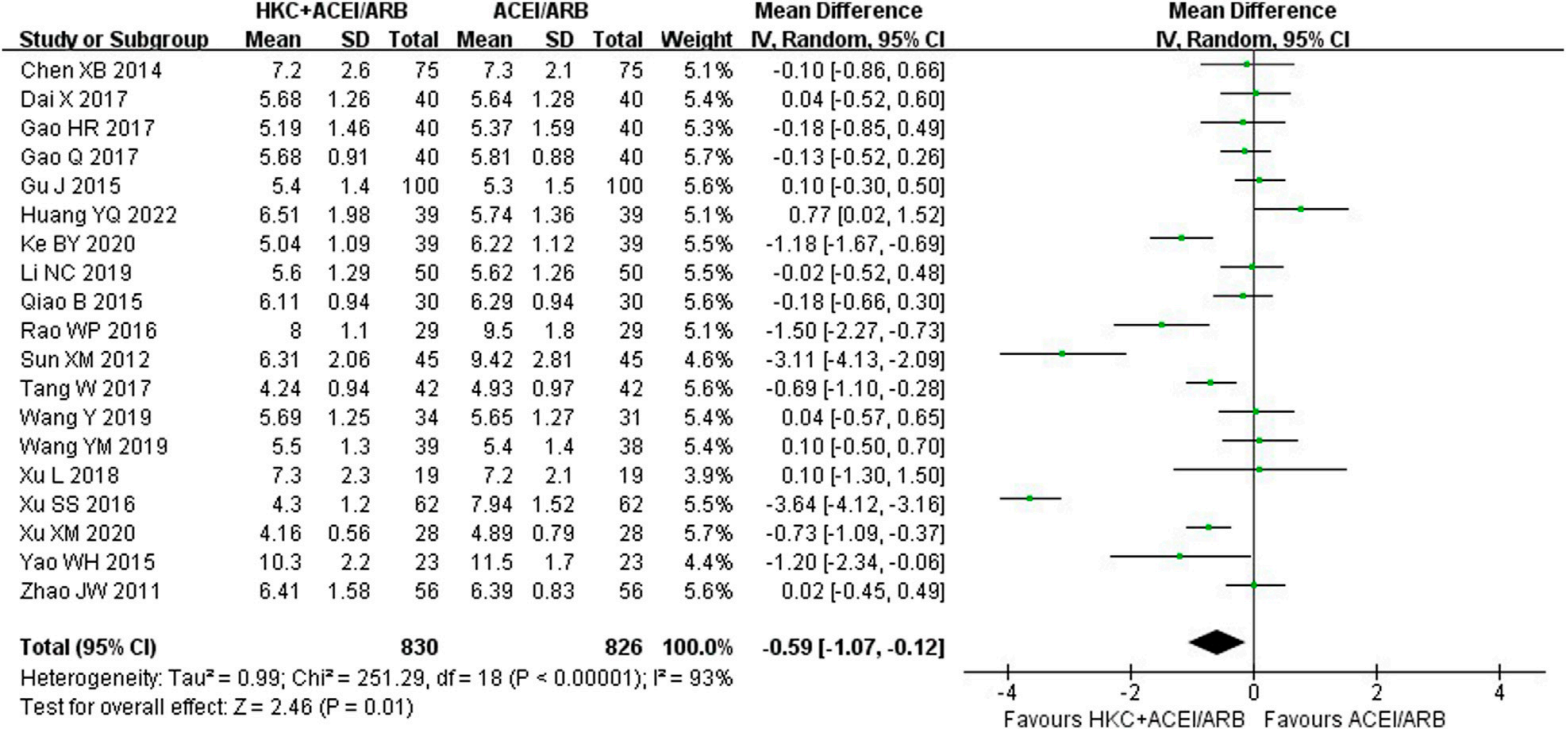
Forest plot of BUN.

In total, nineteen studies (Zhao et al., 2011; Sun et al., 2012; Chen et al., 2014; Qiao 2015a; Gu, 2015; Yao et al., 2015; Rao, 2016; Xu et al., 2016; Dai et al., 2017; Gao, 2017; Gao et al., 2017; Tang, 2017; Xu et al., 2018; Wang 2019a; Wang 2019b; Li, 2019; Ke, 2020; Xu, 2020; Huang et al., 2022) evaluated the BUN levels, involving a total of 1,656 patients. The overall analysis showed that HKC combined with ACEI/ARB was more effective in decreasing BUN than taking ACEI/ARB alone with statistical significance [MD = −0.59, 95% CI (−1.07, −0.12), *p* = 0.01] (Figure 7). Heterogeneity analysis revealed highly significant heterogeneity (I^2^ = 93%, *p* < 0.00001), thus a random-effect model was used for statistical analysis.

The studies were divided into different subgroups according to the treatment duration, the baseline of 24h-UTP, the baseline of Scr and type of RASI (Table 5; Supplementary Figures S13–S16). When stratified by treatment duration, there was no significant difference in decreasing BUN between the experiment group and the control group in the course of treatment at 12 weeks group [MD = −0.38, 95% CI (−0.97, 0.20), *p* = 0.20] or ≥16weeks group [MD = −1.88, 95% CI (−5.32, 1.56), *p* = 0.28], but demonstrated statistical significance compared with the control group in the subgroup analysis of the short-term course of treatment (≤8 weeks) [MD = −0.44, 95% CI (−0.82, −0.06), *p* = 0.02]. Regarding the subgroup analysis based on the baseline of 24h-UTP and Scr, we found that no statistically significant difference was observed in the subgroups of baseline of 24h-UTP 1–2 g [MD = 0.11, 95% CI (−0.13, 0.35), *p* = 0.36], >2 g [MD = −0.94, 95% CI (−1.98, 0.10), *p* = 0.08] and baseline of Scr<90 μmol/L [MD = −0.09, 95% CI (−0.41, 0.23), *p* = 0.58], 90–110 μmol/L [MD = −0.18, 95% CI(−0.58, 0.22), *p* = 0.39] and 110–133 μmol/L [MD = −0.50, 95% CI (−1.13, 0.13), *p* = 0.12]. Furthermore, the statistical significance for the overall effect of BUN was observed (*p* = 0.01), whereas in the subgroup analysis stratified by the type of RASI, no statistical significance was found in the within-group comparison [ACEI: MD = −0.77, 95% CI (−1.59, 0.05), *p* = 0.07; ARB: MD = −0.52, 95% CI (−1.11, 0.07), *p* = 0.09].

**TABLE 5:** Subgroup analyses of BUN based on treatment duration, baseline of 24h-UTP, baseline of Scr and type of RASI.

Criteria for grouping	Subgroups	n	MD (95%CI)	I^2^(%)	Z	P
Treatment duration	8 weeks	10	−0.44(−0.82, −0.06)	74	2.26	0.02
	12 weeks	7	−0.38(−0.97, 0.20)	87	1.28	0.20
	≥16 weeks	2	−1.88(−5.32, 1.56)	99	1.07	0.28
Baseline of 24h-UTP	<1 g	1	−0.69(−1.10, −0.28)	-	3.31	0.0009
	1–2 g	5	0.11(−0.13, 0.35)	0	0.91	0.36
	>2 g	5	−0.94(−1.98, 0.10)	89	1.77	0.08
Baseline of Scr	<90 μmol/L	7	−0.09(−0.41, 0.23)	66	0.56	0.58
	90–110 μmol/L	4	−0.18 (−0.58, 0.22)	61	0.87	0.39
	110–133 μmol/L	5	−0.50 (−1.13, 0.13)	72	1.57	0.12
Type of RASI	ACEI	6	−0.77 (−1.59, 0.05)	87	1.84	0.07
	ARB	13	−0.52 (−1.11, 0.07)	94	1.72	0.09

RASI, Renin-angiotensin-aldosterone system inhibitor; ACEI, Angiotensin-converting enzyme inhibitor; ARB, Angiotensin-receptor blocker; 24h-UTP, 24h-urine total protein; Scr, Serum creatinine.

### 3.8 Safety assessment

**FIGURE 8:**
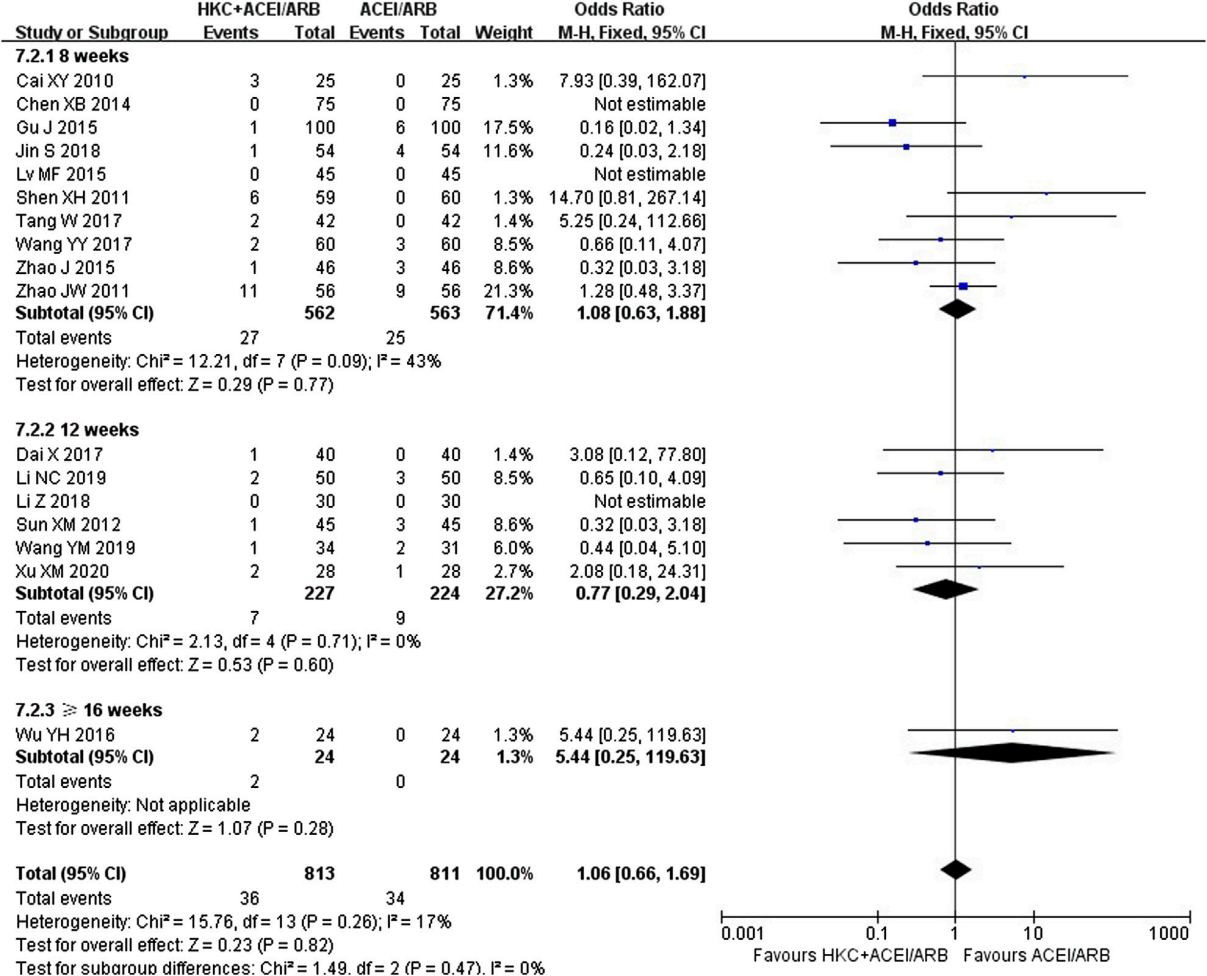
Forest plot of adverse reaction rate.

Seventeen trials (Cai et al., 2010; Shen et al., 2011; Zhao et al., 2011; Sun et al., 2012; Chen et al., 2014; Gu, 2015; Lv and Yu, 2015; Zhao, 2015; Wu, 2016; Dai et al., 2017; Tang, 2017; Wang et al., 2017; Jin and Qin, 2018; Li and Yun, 2018; Wang 2019b; Li, 2019; Xu, 2020) evaluated the safety, covering a total of 1,624 patients. Due to low heterogeneity in these seventeen studies (I^2^ = 17%, *p* = 0.26), a fixed-effect model was utilized. The forest plot illustrated no statistically significant in adverse reaction rate between the HKC+ACEI/ARB group and the ACEI/ARB group [OR = 1.06; 95%CI: (0.66, 1.69), *p* = 0.82] (Figure 8). There were also no significant differences in subgroups based on the treatment duration [≤8weeks: OR = 1.08, 95%CI: (0.63,1.88), *p* = 0.77; 12 weeks: OR = 0.77, 95%CI: (0.29, 2.04), *p* = 0.60; ≥16weeks: OR = 5.44, 95%CI: (0.25, 119.63), *p* = 0.28] and type of RASI [ACEI: OR = 1.00, 95%CI: (0.42, 2.39), *p* = 0.77; ARB: OR = 1.37, 95%CI: (0.83, 2.24), *p* = 0.28] (Supplementary Figure S17). In addition, differences among the subgroup also weren’t found by subgroup analysis based on treatment duration (Test for subgroup differences: I^2^ = 0%, *p* = 0.47) and type of RASI (Test for subgroup differences: I^2^ = 0%, *p* = 0.54). The adverse reaction events reported in the article could be classified into three categories: xerostomia, mild headache or dizziness, and gastrointestinal reactions. One patient in the intervention group (Shen et al., 2011) discontinued the treatment due to intolerance of abdominal distention, and the rest adhered to the treatment during the course. The statistic of adverse reaction events was summarised in Table 6.

**TABLE 6:** Statistic of adverse reaction events.

Adverse reaction events		Intervention group (813 patients)	Control group (811 patients)
Xerostomia		4(0.49%)	11(1.36%)
Mild headache or dizziness		9(1.11%)	14(1.73%)
Gastrointestinal reactions	Mild stomach pain	5(0.62%)	2(0.25%)
	Mild nausea	1(0.12%)	4(0.49%)
	Abdominal distention	13(1.60%)	2(0.25%)
	Diarrhoea	4(0.49%)	1(0.12%)
Total		36(4.43%)	34(4.19%)

### 3.9 Publication bias

**FIGURE 9:**
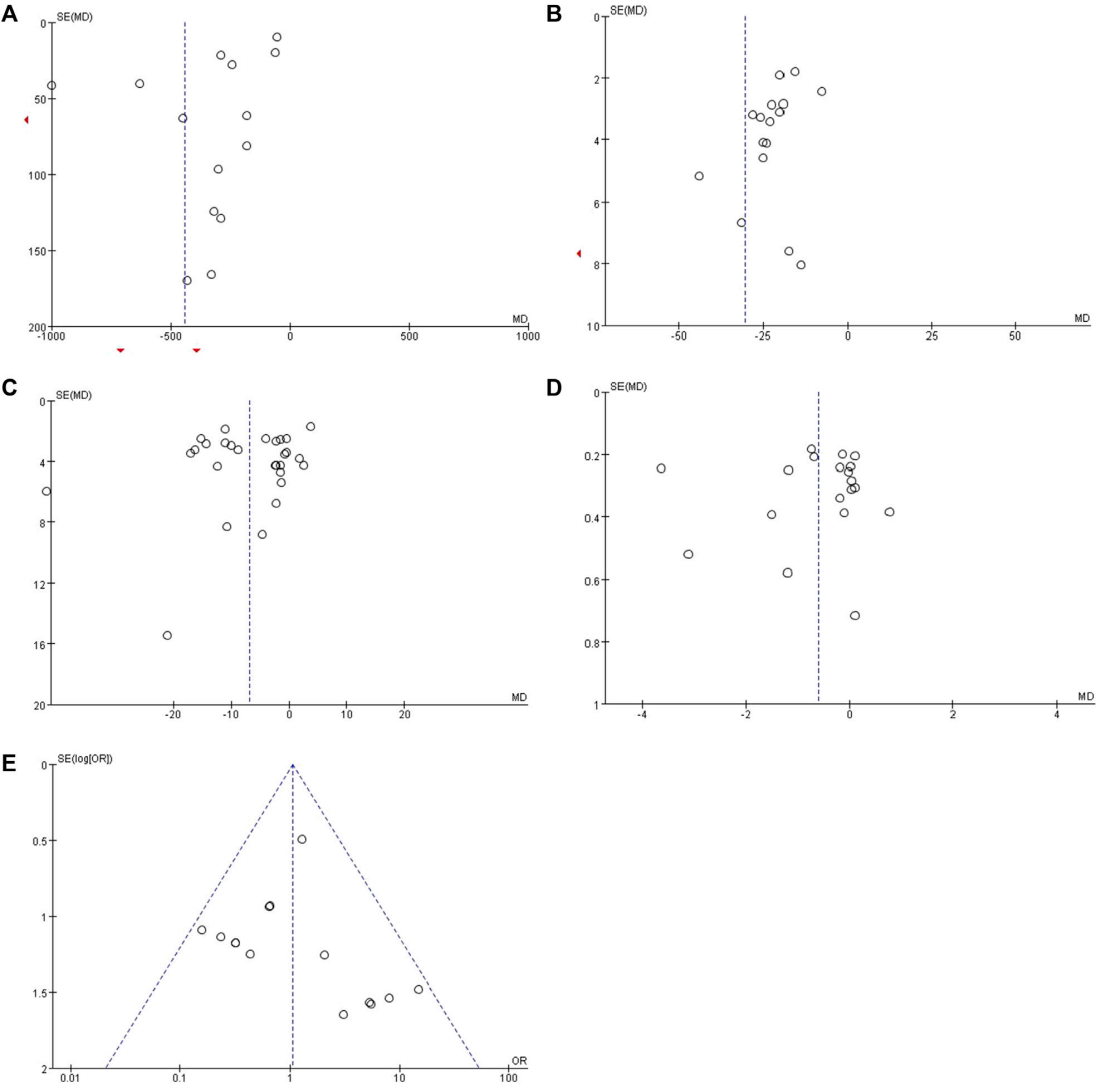
Funnel plots for assessing publication bias. **(A)** 24h-UTP; **(B)** UAER; **(C)** Scr; **(D)** BUN and **(E)** overall adverse reaction rate.

We evaluated publication bias for results from ten or more included studies. The funnel plot drawn for the Scr showed almost symmetry from visual inspection (Figure 9C), which indicated publication bias of the Scr was low. While the funnel plots of 24h-UTP, UAER, BUN and adverse reaction rate showed asymmetry (Figures 9A, B, D, E), indicating that the publication bias possibly existed. As to the UACR, we didn’t evaluate the publication bias because it only included two studies.

## 4 Discussion

In this systematic review, we specifically and comprehensively analyzed the efficacy and safety of the combination of HKC and RASI in treating DKD in the included 32 RCTs composed of 2,881 DKD patients. Our meta-analysis revealed that compared with using ACEI/ARB alone, adding HKC seemed more effective in decreasing 24h-UTP, UAER, UACR, Scr, and BUN without additional adverse reactions. Meanwhile, through subgroup analyses of efficacy indicators based on treatment duration, type of RASI, the baseline of 24h-UTP, and baseline of Scr, we further found that the combination therapy exhibited the exact efficacy in reducing proteinuria. However, the protective effect on renal function could vary depending on the specific circumstances. Moreover, it also seemed that HKC combined with ARB has a more stable and significant therapeutic effect than ACEI. Either way, there was no denying that HKC exhibited significant adjuvant benefits for DKD.

Urinary protein is an essential indicator for the diagnosis and intervention of DKD and a vital independent risk factor that affects the progression of DKD (Neuen et al., 2021). Studies have shown that individuals with proteinuria or albuminuria DKD phenotype have a faster decline in GFR, a more rapid risk of renal failure, and more severe renal pathological changes than those with non-proteinuria or albuminuria DKD in type 1 and type 2 diabetes (Yokoyama et al., 2020; Oshima et al., 2021). Studies have shown that decreasing albuminuria could reduce the risk of renal and cardiovascular endpoint events in DKD patients, and primary renal outcome (ESKD, doubling of serum creatinine, or kidney death) could be reduced by 29% for each 30% decrease in UACR (Oshima et al., 2020). To achieve the ideal control of urinary protein for DKD patients, Chinese physicians often use combination therapy with HKC supplementation. This meta-analysis indicated that HKC combined with RASI exhibited an excellent ability to reduce 24h-UTP, UAER, and UACR in DKD treatment compared to RASI alone. Meanwhile, subgroup results showed that a combination of ARB might be more effective in decreasing UAER than ACEI, and there was no significant difference between the two groups in reducing 24h-UTP when treatment duration ≥16 weeks. It indicated that HKC combined with ARB might have a superior effect on reducing UAER compared to ACEI, but the long-term efficacy still needs further investigation. However, it could not be ruled out that the reason for this result was that the relevant subgroups included only 3 RCTs, respectively. Furthermore, the baseline of 24h-UTP and baseline of Scr may be the central heterogeneity of 24h-UTP and UAER, and treatment duration could be an extra source of heterogeneity on UAER.

Our meta-analysis found that HKC combined with RASI can better reduce Scr, which is consistent with the previous meta-analysis (Shi et al., 2019). What set this meta-analysis apart is that we found the *p*-value on Scr in subgroup analyses of treatment duration ≥16 weeks, baseline of 24h-UTP <1 g or 1–2 g, and baseline of Scr 90–110 μmol/L showed negative results. In a multicenter clinical study on the validation of AM efficacy in 2022, 1,843 patients completed the 24-week course of treatment, and their median baseline of 24-h UTP was 1.122 g. The study showed that combining HKC and RASI reduced proteinuria or albuminuria effectively, but not in Scr (Sun et al., 2022b). It is consistent with our present subgroup analysis, which showed that the subgroup of treatment duration ≥ 16 weeks and baseline of 24h-UTP 1–2 g did not show significant superiority in reducing Scr. It suggests that in terms of decreasing Scr, the combination of HKC and RASI may be more suitable for DKD patients with more proteinuria. In accordance with the prevailing conventional trajectory of DKD (Oshima et al., 2021), it is observed that DKD patients with higher levels of proteinuria often experience a moderate to severe decline in renal function. As for the DKD patients who already exhibited impaired renal function, our subgroup analysis on Scr indicated that those with a baseline Scr level ranging from 110–133 μmol/L and exceeding 133 μmol/L demonstrated greater efficacy in reducing Scr compared to those with a baseline Scr level of 90–110 μmol/L. Furthermore, in the real-world clinical setting, many DKD patients go to the hospital only when they notice overt proteinuria or a marked decline in renal function, which can be called the ‘silent crowd effect’ (Szczech et al., 2014; Gembillo et al., 2021). Moreover, this type of patient is also the focus and difficulty of current clinical treatment, and the effect of Western medicine treatment is not significant. The results of our meta-analysis may provide a novel and effective treatment strategy and convincing evidence for such DKD patients. Although adding HKC showed a superior effect in decreasing BUN, no significant differences were observed in multiple subgroups. Considering that BUN was easily affected by various factors, it merely served as an auxiliary reminder for renal function. More rigorous and in-depth clinical studies are needed to confirm in the future. In addition, the sources of the central heterogeneity of Scr and BUN come from the baseline of 24h-UTP and the baseline of Scr.

Various factors are involved in the pathogenesis of DKD, including metabolic disorders, hemodynamic abnormalities, inflammatory responses, oxidative stress, and epigenetics (Ilyas et al., 2017). Research has found that for chronic progressive kidney injury, regardless of the initiating factors and signaling pathways, the main renal pathological changes caused by it are inflammation and fibrosis, which interact with each other and ultimately lead to proteinuria and loss of renal function (Kanasaki et al., 2013; Hung et al., 2021). DCCT/EDIC and UKPDS follow-up studies have shown that patients with poor early blood sugar control, even with subsequent intensive hypoglycemic treatment, still cannot block the occurrence and development of DKD, indicating the existence of adverse “metabolic memory” effect, which is considered an essential reason for contributing to the progression of renal fibrosis (Zheng et al., 2021). Meanwhile, epigenetic modification is generally accepted as an essential mechanism for adverse “metabolic memory” in academia (Zheng et al., 2021). Therefore, finding effective drugs to control renal inflammation and fibrosis and block the progression of renal fibrosis mediated by adverse “metabolic memory” effects by regulating epigenetics may be an essential breakthrough in preventing and treating DKD. RASI treats DKD mainly by improving hemodynamics, but it does not have anti-inflammatory and anti-fibrotic effects nor reverse epigenetic modification, so its clinical efficacy is limited to some extent (Reddy et al., 2013). In the AM, seven flavonoids have been confirmed as significant pharmacologically bioactive constituents, including Hyperoside, Hibifolin, Rutin, Myricetin, Isoquercetin, Quercetin, and Quercetin-3-O-robinobioside, which can be called TFA (Li et al., 2021). TFA can improve renal inflammatory fibrosis and reduce proteinuria and creatinine levels in DKD models *in vitro* and *in vivo* by inhibiting the expression of the endoplasmic reticulum stress-activated iRhom2/TACE system and its mediated post-translational inflammatory and fibrotic factors, such as TNFα, TGF-β, α-S MA, and collagen IV (Cai et al., 2017; Liu et al., 2017). Some studies also found that TFA could exert a protective effect in kidney injury by inhibiting ferroptosis and reducing the inflammatory response by activating Nrf and scavenging reactive oxygen species (ROS) (Wang et al., 2023). In the realm of epigenetic modification, it has been demonstrated that a variety of transcriptional/post-translational modifications (such as acetylation, deacetylation, methylation, and microRNAs) in the high blood sugar environment have a “metabolic memory” nature and that the epigenetic alterations caused by these modifications can lead to dysregulation of relevant gene expression, resulting in the chronic inflammatory and fibrotic damage to the kidney (Zheng et al., 2021). At the transcriptional level, TFA can target and upregulate the activity of demethylase ALKBH5, reverse Snail m6A methylation-mediated EMT, and play an anti-fibrotic role in DKD (Ning et al., 2020). Additionally, TFA can also inhibit histone acetyltransferase p300 (HAT p300), reducing the degree of acetylation of histone H3, thereby alleviating the undesirable “metabolic memory” effect. Subsequently, this mechanism can inhibit the transcriptional activity of the components of the extracellular matrix and downregulate the expression of the linker proteins fibronectin, laminin, and collagen, thereby reducing renal inflammation and fibrosis (Ramaiah et al., 2021; Nowrasteh et al., 2023). At the translational level, TFA can directly inhibit the activity of the renal fibrosis-promoting histone deacetylase 3 (HDAC3) and activate the action of the renal fibrosis-inhibiting SIRT1, thereby negatively regulating the process of renal fibrosis, slowing down the onset and development of DKD (Huang et al., 2016; Liu et al., 2020). Because of the above-mentioned active ingredients, HKC not only can reduce the inflammatory response, inhibit renal fibrosis, combat oxidative stress, protect renal tubular epithelial cells, and inhibit ferroptosis through multiple targets and pathways (Li et al., 2021), but also can reverse adverse “metabolic memory” effects mediated by epigenetic modifications (Kim et al., 2016; Li et al., 2021), although there is no substantial evidence to suggest that HKC can effectively induce haemodynamic improvement. Thus, the combination of HKC and RASI can optimize the deficiency of each other, enabling a more comprehensive and effective treatment for DKD patients, which may be the reason for the significant therapeutic efficacy of the addition of HKC.

Despite the comprehensive nature of this meta-analysis, certain inevitable limitations within this meta-analysis need to be taken into further consideration. Firstly, some studies did not adequately define their randomization process or explicitly state whether allocation concealment and blinding were implemented, which could impact the accuracy and reliability of the analysis results. Secondly, the language of the included studies was limited to only Chinese and English, potentially leading to the selection bias. Furthermore, given that HKC are primarily used in China, most clinical studies on this subject have also been conducted in China, leading to most of the relevant articles being written in Chinese. Although all the included articles can be traced in the Chinese official databases, many of them were not accessible in international databases or published in non-indexed journals, which was also a common challenge faced by many traditional Chinese medicines. Therefore, the above issues may potentially affect the quality of the included RCTs to some extent, and we need to be cautious in explaining the results obtained. Thirdly, the included RCTs had a relatively short duration of treatment, with most studies only lasting a few weeks to a few months. Only one study had a duration that went up to 1 year. Therefore, the number of articles ≥ 16 weeks was low in all subgroup analyses regarding duration of treatment, which might account for the lack of statistically significant differences in outcome indicators in subgroups with longer duration of treatment. Additionally, no follow-up assessments were conducted at the end of the intervention, limiting the ability to observe long-term and dosage effects. Last but not least, a limited amount of literature is available regarding renal endpoints after AM intervention. We opted to use indicators such as 24h-UTP, UAER, UACR, Scr, and BUN to evaluate renal function comprehensively; however, there are still significant limitations in using these indicators as alternative endpoints for assessing the progression of DKD. Therefore, it is essential for researchers to interpret the results of this meta-analysis with caution and to conduct further studies with double-blindness, allocation concealment, multi-centered, and reporting negative results to address these limitations.

Despite the abovementioned limitations, this systematic review and meta-analysis provide valuable insights. To the best of our knowledge, it is the most comprehensive and up-to-date systematic review and meta-analysis of the efficacy and safety of HKC combined with RASI in the treatment of DKD, with subgroups analyzed according to treatment duration, type of RASI, the baseline of 24h-UTP and Scr. It is also the first systematic review and meta-analysis to explore whether HKC combined with ACEI or ARB treatment for DKD has similar efficacy and safety. The latest meta-analysis on the combination of HKC and RASI in treating DKD was conducted in 2019 (Shi et al., 2019), and since then, there have been new relevant clinical studies. What sets this meta-analysis apart is that it includes more comprehensive subgroup analysis and safety assessment compared to previous meta-analyses, which may provide more valuable guidance for clinical application. In 2022, a meta-analysis compared Tripterygium glycoside tablets (TG) in combination with AM to the use of AM alone for the treatment of DKD. However, the results of this study indicated a significant increase in adverse reactions when AM was combined with TG. This elevation in adverse events may pose challenges for patients with chronic conditions, such as DKD, who require long-term medication. Furthermore, among the three efficacy indicators studied in that research, there remained a need for further research to substantiate the efficacy of the combination therapy in preserving kidney function. Our study, as compared to the meta-analysis mentioned above, selected RASI, which was more widely used in clinical practice and recommended by DKD guidelines, as the control group. Furthermore, our study conducted subgroup analyses on all five efficacy indicators from four different perspectives, aiming to comprehensively discuss the effectiveness and safety of combining AM with RASI in treating DKD. Additionally, our study innovatively explored whether there were differences in the treatment of proteinuria and kidney function in DKD when AM was combined with ACEI or when it was combined with ARB, starting from the perspective of the type of RASI. Last but not least, our paper also delved into the mechanistic aspect, analyzing why the combination of AM and RASI was more effective in treating DKD, specifically, why it was particularly suitable for DKD patients with higher levels of 24h-UTP or elevated baseline of Scr. These results provided valuable insights for further in-depth research on AM and might offer new therapeutic perspectives for clinical physicians. Notably, the meta-analysis on the combination of TG and AM mentioned above did not address these aspects.

## 5 Conclusion

The results of the systematic review and meta-analysis indicate that the combination of HKC and RASI is significantly more effective in reducing urinary protein than using RASI alone, and the combination with ARB may be more effective than the combination with ACEI. The addition of AM may have a more significant clinical effect in decreasing Scr for DKD patients with 24h-UTP > 2 g or Scr levels > 110–133 μmol/L and > 133 μmol/L. In terms of safety, all treatment durations demonstrate good safety, and adding HKC to either ACEI or ARB did not lead to additional side effects. However, due to the high clinical heterogeneity and non-standardized nature of the included trials, high-quality clinical RCTs at different stages of DKD are needed in the future to confirm the current results.

## Data Availability

The original contributions presented in the study are included in the article/Supplementary Material, further inquiries can be directed to the corresponding author.

## Abbreviations

DKD, Diabetic kidney disease; CKD, Chronic kidney disease; AM, Abelmoschus Manihot; RASI, Renin-angiotensin-aldosterone system inhibitor; RCTs, Randomized control trials; TCM, Traditional Chinese medicine; DM, Diabetes mellitus; ESKD, End-stage kidney disease; ACEIs, Angiotensin-converting enzyme inhibitors; ARBs, Angiotensin-receptor blockers; SGLT2i, Sodium-glucose cotransporter 2 inhibitor; HKC, Huangkui capsule; 24h-UTP, 24h-urine total protein; UAER, Urinary albumin excretion rates; UACR, Urinary albumin/creatinine ratio; Scr, Serum creatinine; BUN, blood urea nitrogen; BT, basic treatment; ORs, Odds ratios; Cis, Confidence intervals.
