# Supplementary figures and images for: Efficacy and safety of Abelmoschus manihot capsule combined with ACEI/ARB on diabetic kidney disease: a systematic review and meta analysis
Source: Front Pharmacol. 2024 Jan 5;14:1288159. doi: 10.3389/fphar.2023.1288159 (PMC10796716; doi:10.3389/fphar.2023.1288159)

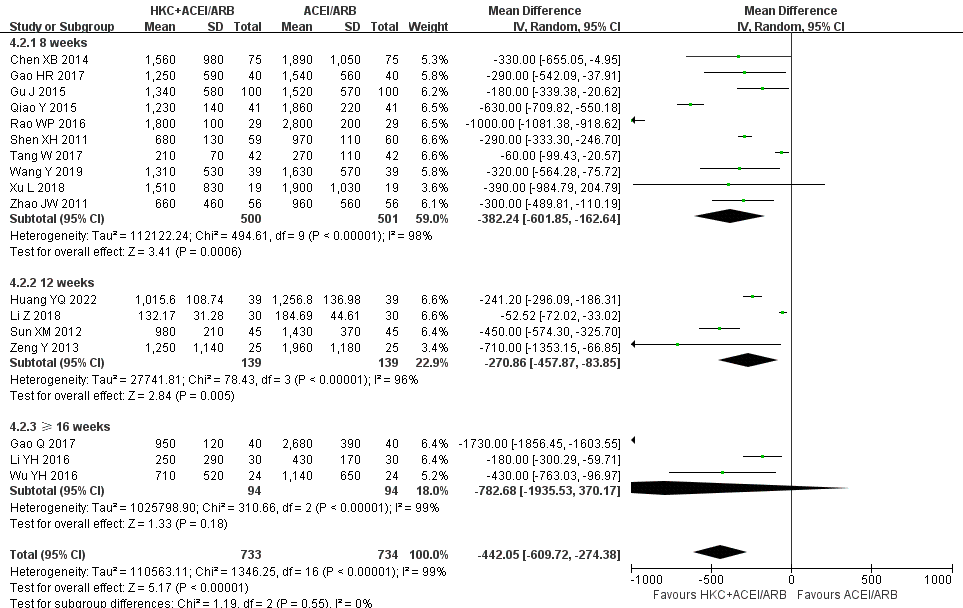

Supplement: Supplementary file 2 [file DataSheet1.ZIP › Supplementary Figures S1-17/Supplementary Figure S1(revised).png]

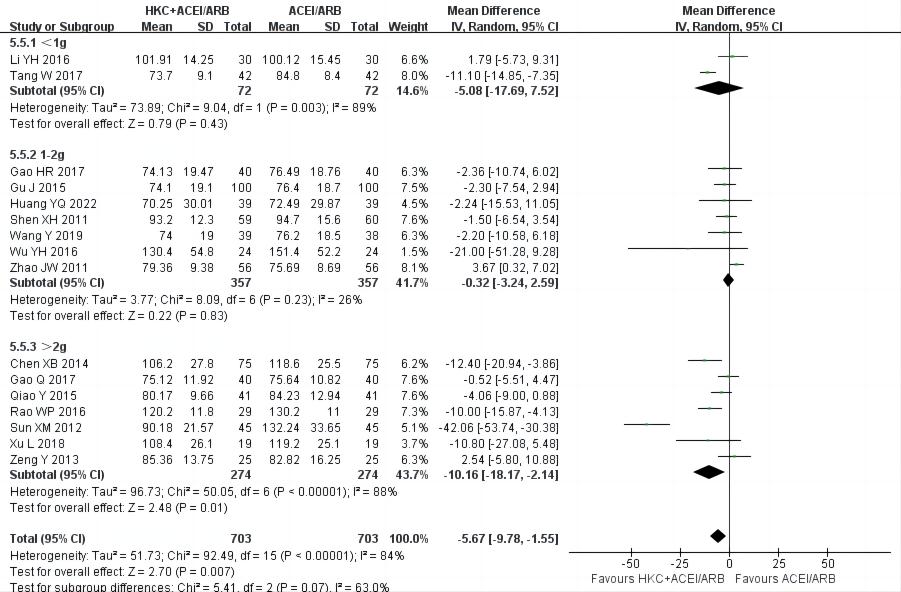

Supplement: Supplementary file 2 [file DataSheet1.ZIP › Supplementary Figures S1-17/Supplementary Figure S10.jpg]

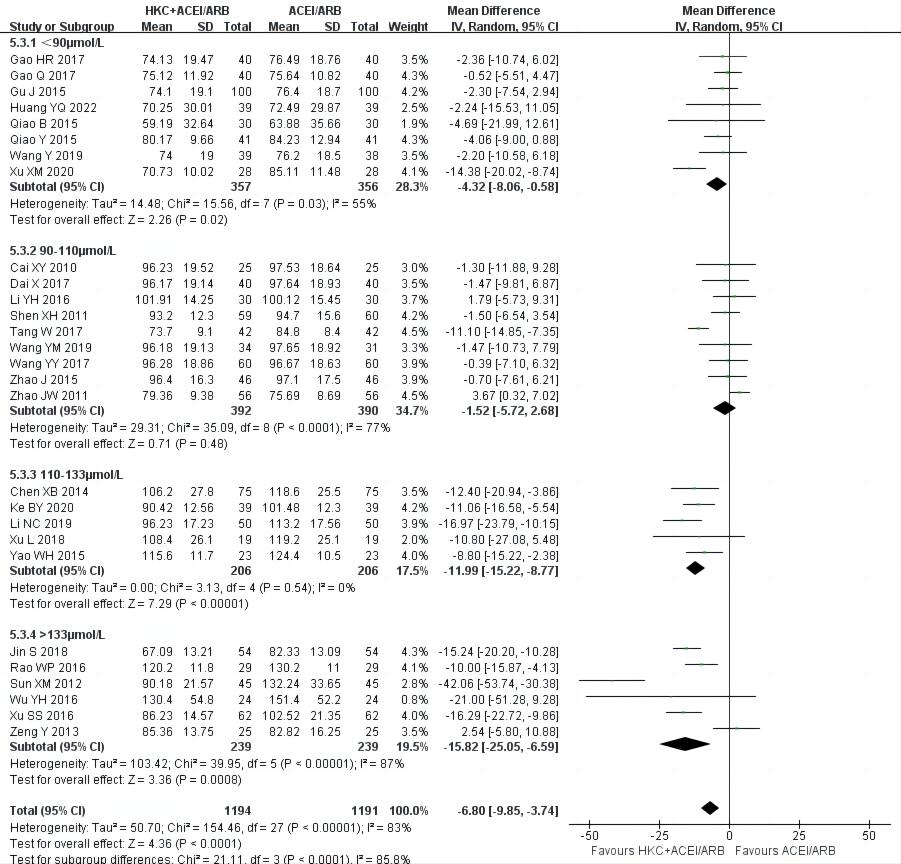

Supplement: Supplementary file 2 [file DataSheet1.ZIP › Supplementary Figures S1-17/Supplementary Figure S11.jpg]

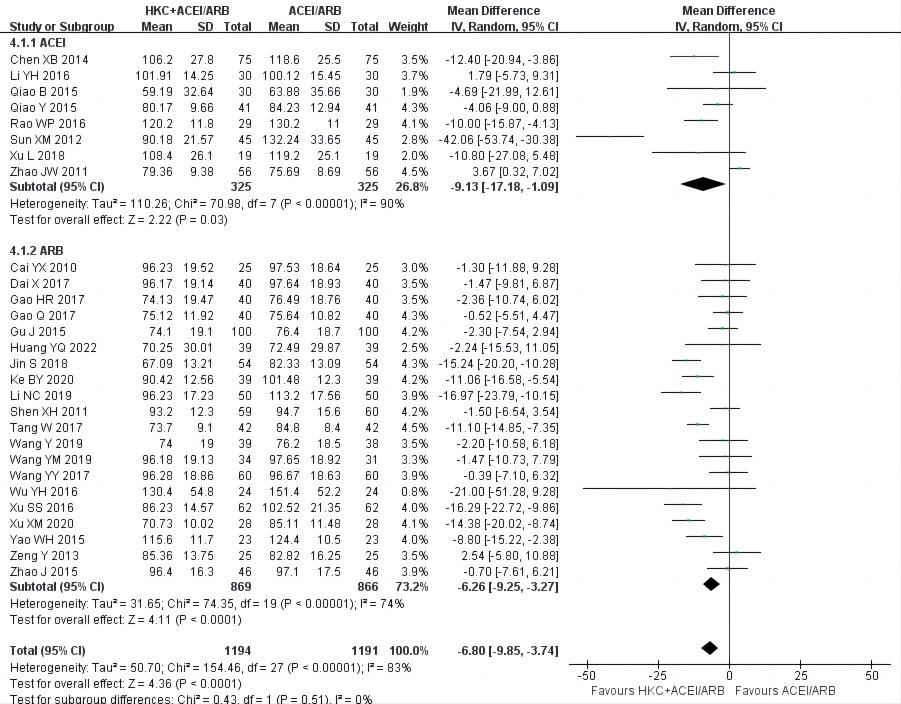

Supplement: Supplementary file 2 [file DataSheet1.ZIP › Supplementary Figures S1-17/Supplementary Figure S12.jpg]

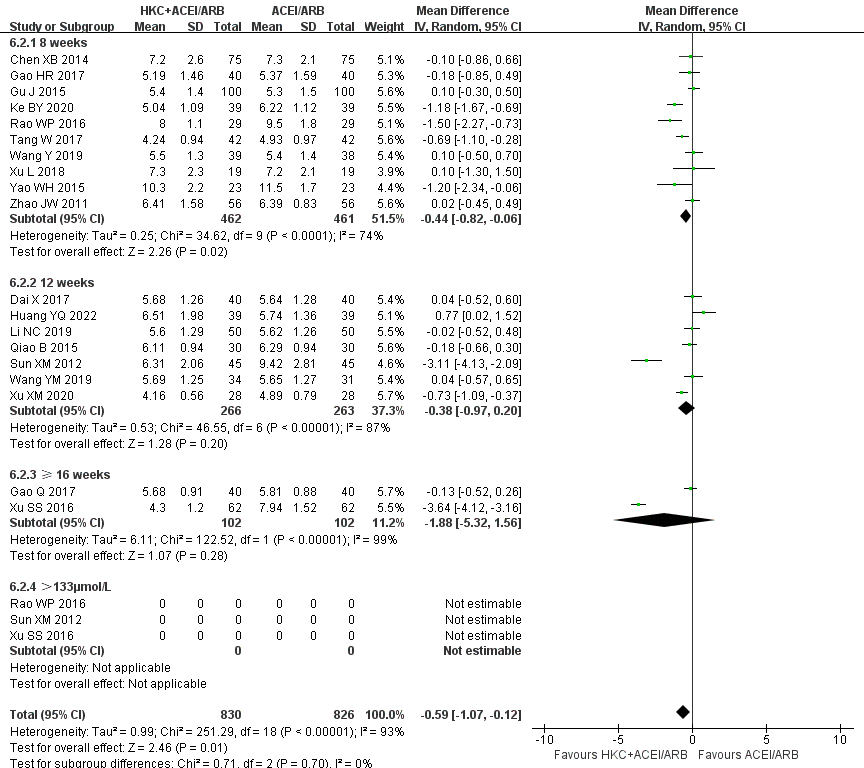

Supplement: Supplementary file 2 [file DataSheet1.ZIP › Supplementary Figures S1-17/Supplementary Figure S13(revised).png]

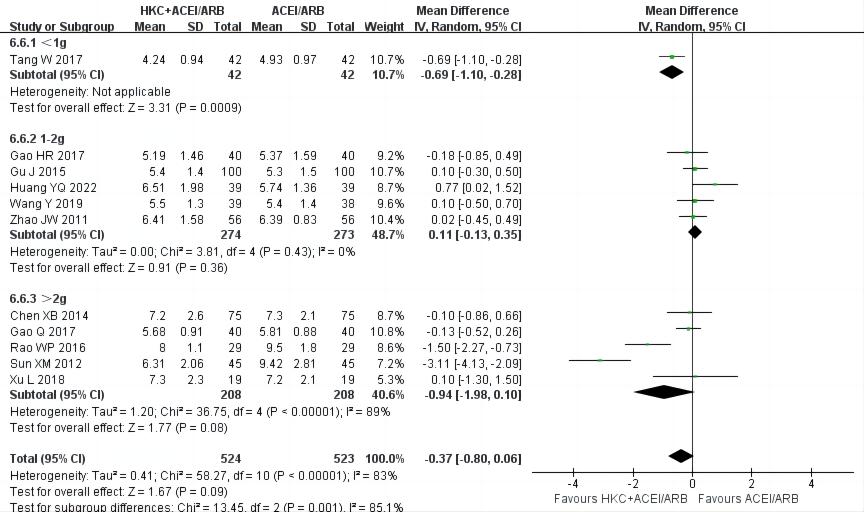

Supplement: Supplementary file 2 [file DataSheet1.ZIP › Supplementary Figures S1-17/Supplementary Figure S14.jpg]

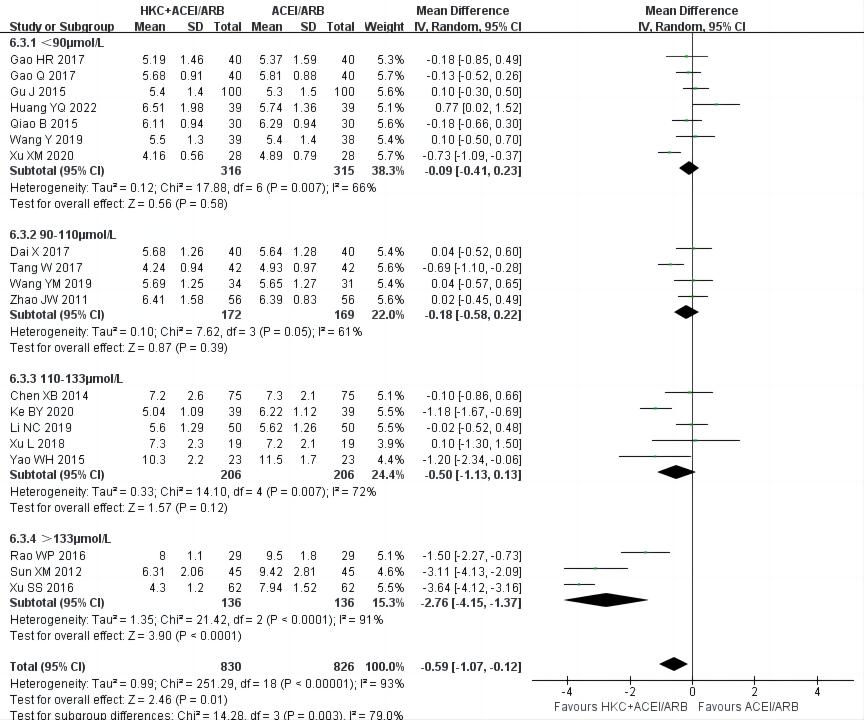

Supplement: Supplementary file 2 [file DataSheet1.ZIP › Supplementary Figures S1-17/Supplementary Figure S15.jpg]

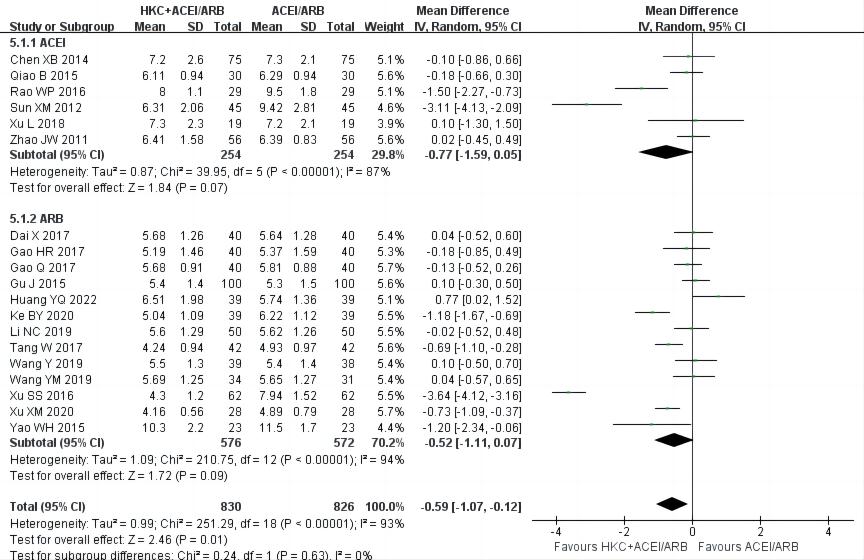

Supplement: Supplementary file 2 [file DataSheet1.ZIP › Supplementary Figures S1-17/Supplementary Figure S16.jpg]

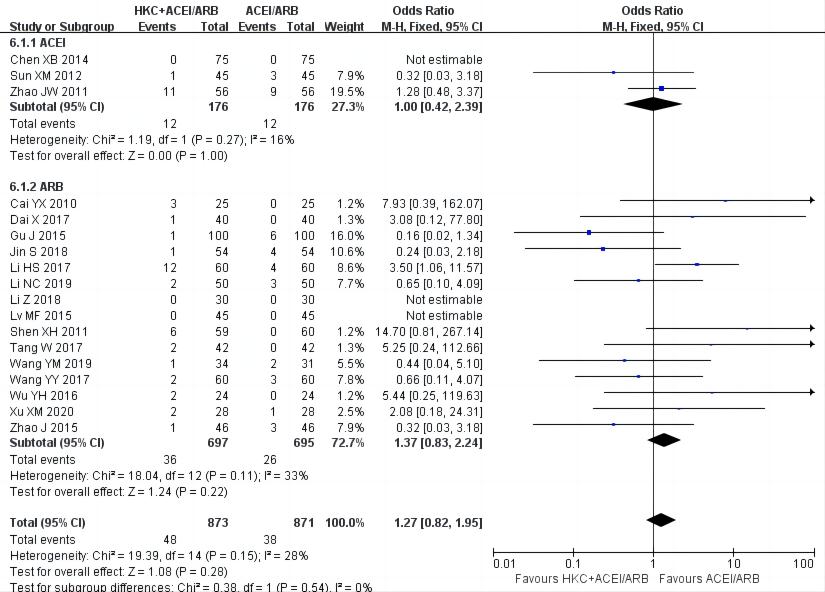

Supplement: Supplementary file 2 [file DataSheet1.ZIP › Supplementary Figures S1-17/Supplementary Figure S17.jpg]

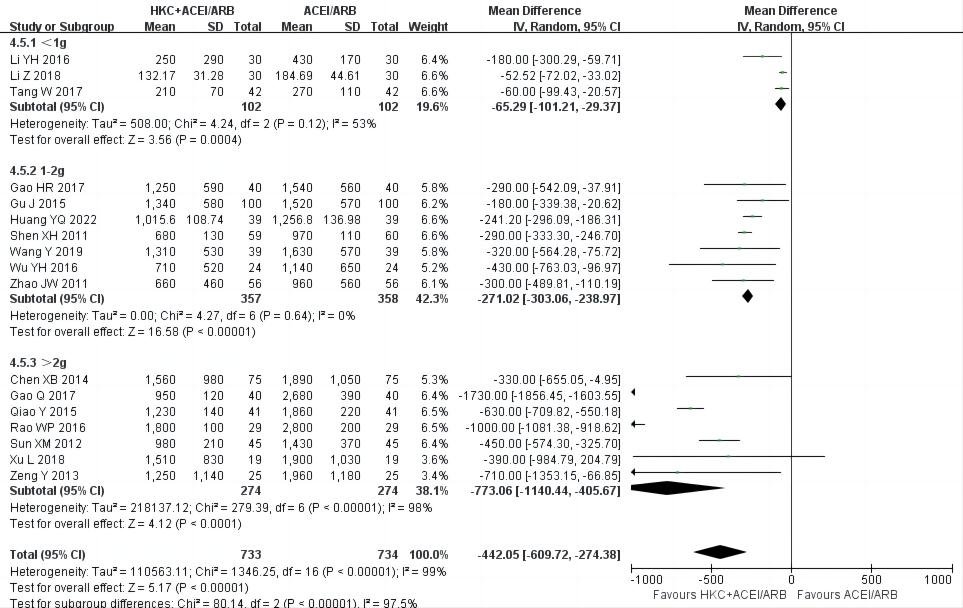

Supplement: Supplementary file 2 [file DataSheet1.ZIP › Supplementary Figures S1-17/Supplementary Figure S2.jpg]

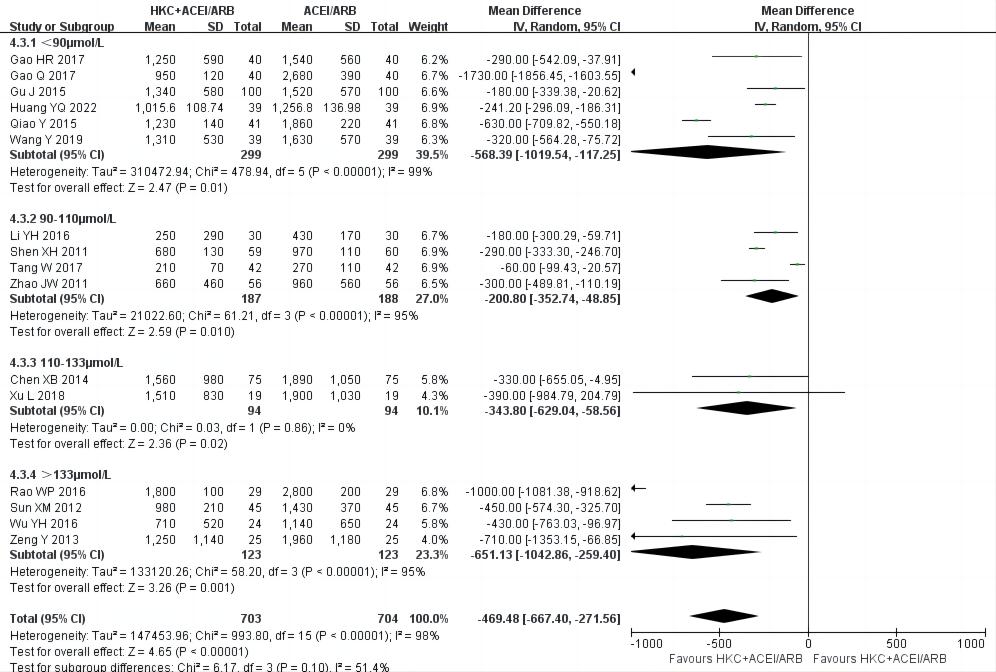

Supplement: Supplementary file 2 [file DataSheet1.ZIP › Supplementary Figures S1-17/Supplementary Figure S3.jpg]

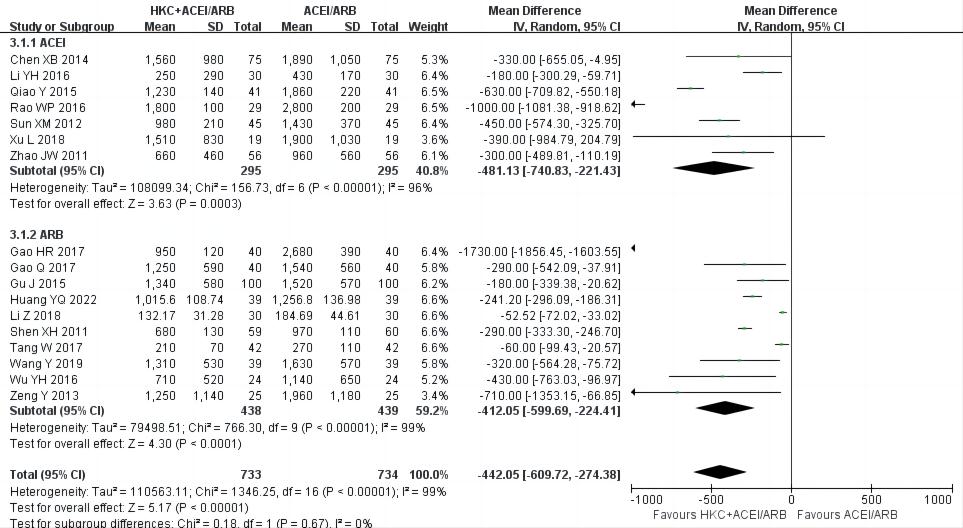

Supplement: Supplementary file 2 [file DataSheet1.ZIP › Supplementary Figures S1-17/Supplementary Figure S4.jpg]

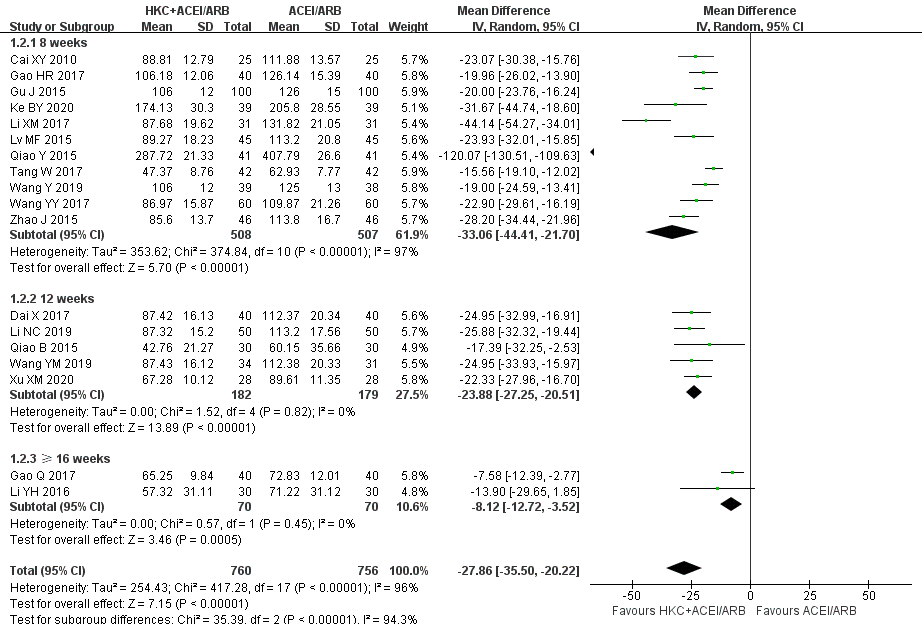

Supplement: Supplementary file 2 [file DataSheet1.ZIP › Supplementary Figures S1-17/Supplementary Figure S5(revised).png]

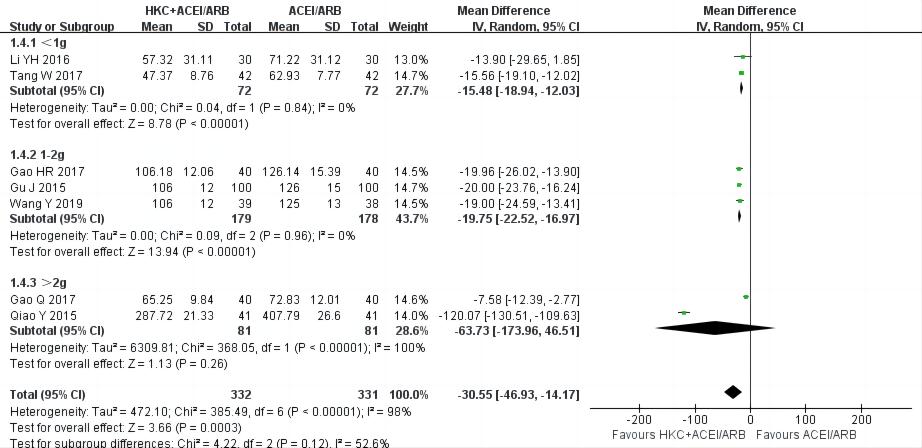

Supplement: Supplementary file 2 [file DataSheet1.ZIP › Supplementary Figures S1-17/Supplementary Figure S6.jpg]

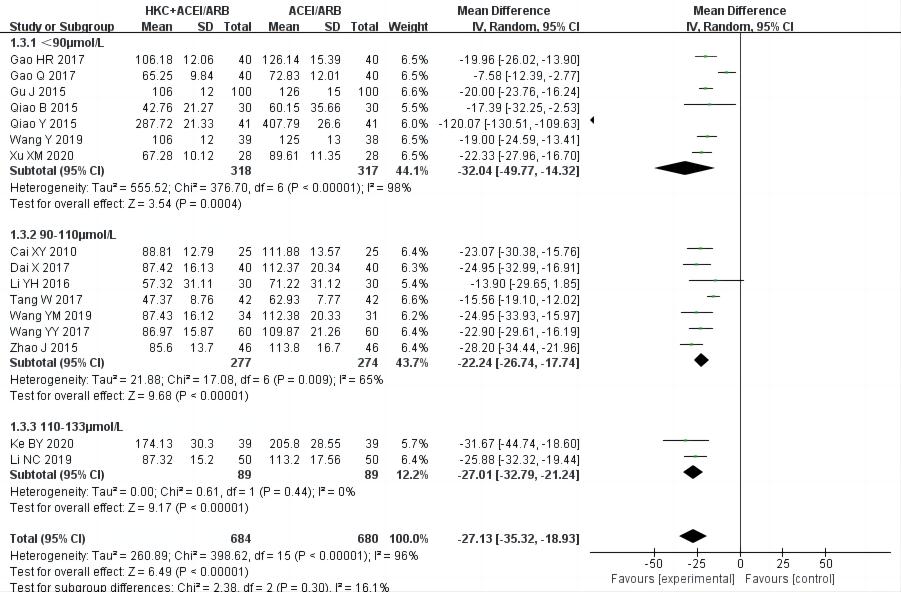

Supplement: Supplementary file 2 [file DataSheet1.ZIP › Supplementary Figures S1-17/Supplementary Figure S7.jpg]

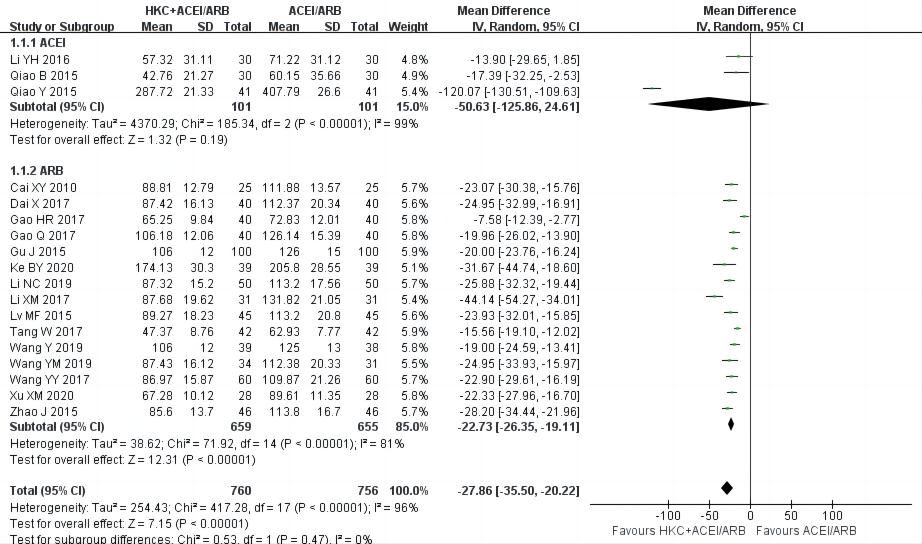

Supplement: Supplementary file 2 [file DataSheet1.ZIP › Supplementary Figures S1-17/Supplementary Figure S8.jpg]

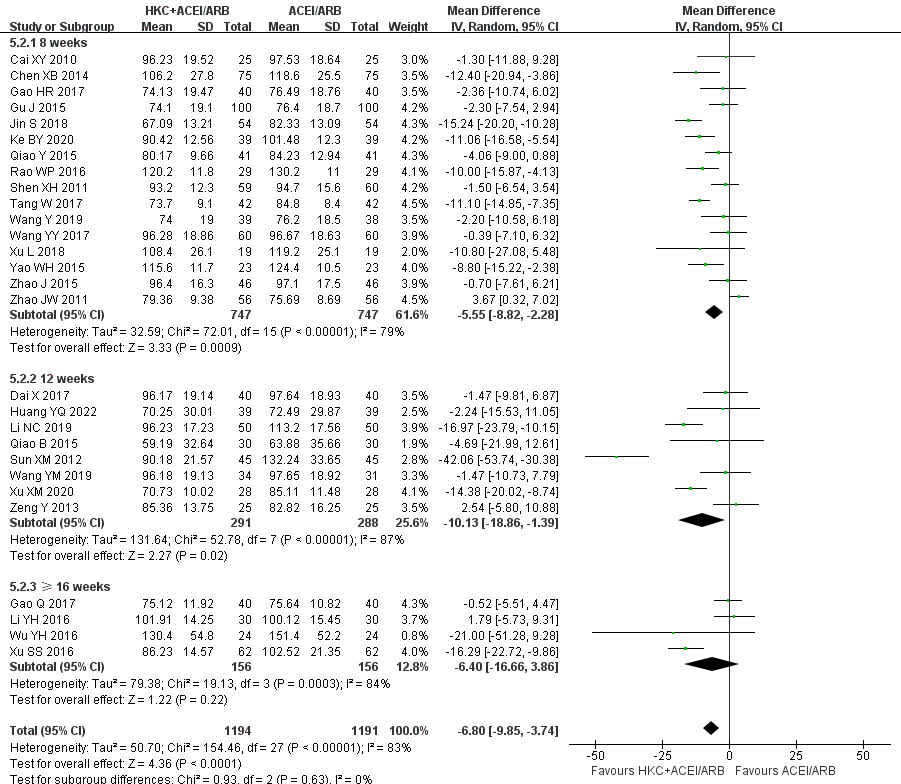

Supplement: Supplementary file 2 [file DataSheet1.ZIP › Supplementary Figures S1-17/Supplementary Figure S9(revised).png]
