# Supplementary material for: Efficacy and safety of Abelmoschus manihot capsule combined with ACEI/ARB on diabetic kidney disease: a systematic review and meta analysis
Source: Front Pharmacol. 2024 Jan 5;14:1288159. doi: 10.3389/fphar.2023.1288159 (PMC10796716; doi:10.3389/fphar.2023.1288159)
Supplement: Supplementary file 3 [file Table2.docx]

**Supplementary Table 1:**

**Search strategy in CNKI**： (黄葵胶囊 + 黄葵) * 糖尿病肾病

**Search strategy in Wanfang Database**：(黄葵胶囊 or 黄葵）and 糖尿病肾病

**Search strategy in VIP：** (黄葵胶囊 + 黄葵) * 糖尿病肾病

**Search strategy in SinoMed：**("黄葵胶囊"[常用字段] OR "黄葵"[常用字段]) AND ("糖尿病肾病"[常用字段] OR "糖尿病性肾小球硬化症"[常用字段] OR "糖尿病肾病"[主题词])

**Search strategy in PubMed**

1. "Abelmoschus OR Abelmoschus esculentus OR Abelmoschus manihot OR Flos Abelmoschus manihot OR Abelmoschi corolla OR Okra OR Okras OR ambrette OR Huangkui OR Huangshukui OR Huangshukuihua" [Text Word]

2. "Diabetic Nephropathies or Nephropathies, Diabetic or Nephropathy, Diabetic or Diabetic Nephropathy or Diabetic Kidney Disease or Diabetic Kidney Diseases or Kidney Disease, Diabetic or Kidney Diseases, Diabetic or Diabetic Glomerulosclerosis or Glomerulosclerosis, Diabetic or Intracapillary Glomerulosclerosis or Nodular Glomerulosclerosis or Glomerulosclerosis, Nodular or Kimmelstiel-Wilson Syndrome or Kimmelstiel Wilson Syndrome or Syndrome, Kimmelstiel-Wilson or Kimmelstiel-Wilson Disease or Kimmelstiel Wilson Disease" [Text Word]

3. 1 and 2

**Search strategy in EMBASE**

1.'Diabetic Nephropathies':ab,ti OR 'Kimmelstiel Wilson Syndrome':ab,ti OR 'Nodular Glomerulosclerosis':ab,ti OR 'Kimmelstiel Wilson Disease':ab,ti OR 'Syndrome, Kimmelstiel-Wilson ':ab,ti OR 'Kimmelstiel-Wilson Syndrome':ab,ti OR 'Glomerulosclerosis, Nodular':ab,ti OR 'Intracapillary Glomerulosclerosis':ab,ti OR 'Kimmelstiel-Wilson Disease':ab,ti OR 'Nephropathy, Diabetic':ab,ti OR 'Diabetic Nephropathy':ab,ti OR 'Kidney Disease, Diabetic':ab,ti OR 'Diabetic Kidney Disease':ab,ti OR 'Diabetic Kidney Diseases':ab,ti OR 'Kidney Diseases, Diabetic':ab,ti OR 'Nephropathies, Diabetic':ab,ti OR 'Diabetic Glomerulosclerosis':ab,ti OR 'Glomerulosclerosis, Diabetic'ab,ti

2. Abelmoschus:ab,ti OR Abelmoschus esculentus:ab,ti OR Abelmoschus manihot:ab,ti OR Flos Abelmoschus manihot:ab,ti OR Abelmoschi corolla:ab,ti OR Okra:ab,ti OR Okras:ab,ti OR ambrette:ab,ti OR Huangkui:ab,ti OR Huangshukui:ab,ti OR Huangshukuihua:ab,ti

3. 1 AND 2

**Search strategy in Cochrane Library**

1. "Abelmoschus OR Abelmoschus esculentus OR Abelmoschus manihot OR Flos Abelmoschus manihot OR Abelmoschi corolla OR Okra OR Okras OR ambrette OR Huangkui OR Huangshukui OR Huangshukuihua"[All Text]

2. "Diabetic Nephropathies or Nephropathies, Diabetic or Nephropathy, Diabetic or Diabetic Nephropathy or Diabetic Kidney Disease or Diabetic Kidney Diseases or Kidney Disease, Diabetic or Kidney Diseases, Diabetic or Diabetic Glomerulosclerosis or Glomerulosclerosis, Diabetic or Intracapillary Glomerulosclerosis or Nodular Glomerulosclerosis or Glomerulosclerosis, Nodular or Kimmelstiel-Wilson Syndrome or Kimmelstiel Wilson Syndrome or Syndrome, Kimmelstiel-Wilson or Kimmelstiel-Wilson Disease or Kimmelstiel Wilson Disease" [All Text]

3. 1 and 2
